# Molecular Evolution of Tryptophan Hydroxylases in Vertebrates: A Comparative Genomic Survey

**DOI:** 10.3390/genes10030203

**Published:** 2019-03-08

**Authors:** Junmin Xu, Yanping Li, Yunyun Lv, Chao Bian, Xinxin You, Daiji Endoh, Hiroki Teraoka, Qiong Shi

**Affiliations:** 1 School of Veterinary Medicine, Rakuno Gakuen University, Ebetsu 069-8501, Japan; xujunmin@genomics.cn (J.X.); dendoh@rakuno.ac.jp (D.E.); 2 BGI-Shenzhen, Shenzhen 518083, China; liyanping@genomics.cn; 3 Shenzhen Key Lab of Marine Genomics, Guangdong Provincial Key Lab of Molecular Breeding in Marine Economic Animals, BGI Academy of Marine Sciences, BGI Marine, BGI, Shenzhen 518083, China; lvyunyun@genomics.cn (Y.L.); bianchao@genomics.cn (C.B.); youxinxin@genomics.cn (X.Y.); 4 BGI Education Center, University of Chinese Academy of Sciences, Shenzhen 518083, China

**Keywords:** tryptophan hydroxylase (TPH), serotonin, melatonin biosynthesis, phylogenetic analysis, molecular evolution, positive selection, vertebrate

## Abstract

Serotonin is a neurotransmitter involved in various physiological processes in the central and peripheral nervous systems. Serotonin is also a precursor for melatonin biosynthesis, which mainly occurs in the pineal gland of vertebrates. Tryptophan hydroxylase (TPH) acts as the rate-limiting enzyme in serotonin biosynthesis and is the initial enzyme involved in the synthesis of melatonin. Recently, two enzymes—TPH1 and TPH2—were reported to form the TPH family in vertebrates and to play divergent roles in serotonergic systems. Here, we examined the evolution of the TPH family from 70 vertebrate genomes. Based on the sequence similarity, we extracted 184 predicted *tph* homologs in the examined vertebrates. A phylogenetic tree, constructed on the basis of these protein sequences, indicated that *tph* genes could be divided into two main clades (*tph1* and *tph2*), and that the two clades were further split into two subgroups of tetrapods and Actinopterygii. In tetrapods, and some basal non-teleost ray-finned fishes, only two *tph* isotypes exist. Notably, *tph1* in most teleosts that had undergone the teleost-specific genome duplication could be further divided into *tph1a* and *tph1b*. Moreover, protein sequence comparisons indicated that TPH protein changes among vertebrates were concentrated at the NH_2_-terminal. The tertiary structures of TPH1 and TPH2 revealed obvious differences in the structural elements. Five positively selected sites were characterized in TPH2 compared with TPH1; these sites may reflect the functional divergence in enzyme activity and substrate specificity. In summary, our current work provides novel insights into the evolution of *tph* genes in vertebrates from a comprehensive genomic perspective.

## 1. Introduction

**Figure 1:**
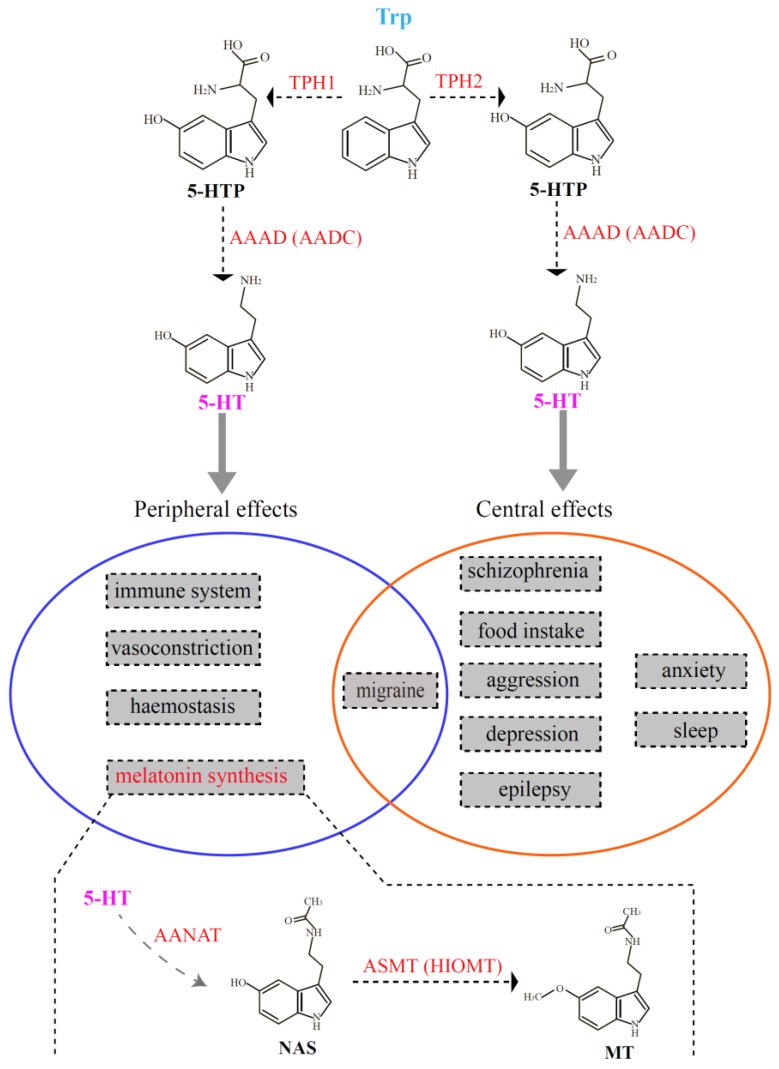
Schematic representation of the two serotonin systems in vertebrates. The top part denotes the process of serotonin biosynthesis, which is also the first two steps in melatonin synthesis. The middle part (in the circles) summarizes the functions regulated by the two serotonin systems. The bottom part interprets the last two steps for melatonin synthesis. 5-HT, 5-hydroxytryptamine; AAAD (AADC), l-aromatic amino acid decarboxylase; AANAT, aralkylamine *N*-acetyltransferase; ASMT, acetylserotonin-*O*-methyltransferase; HIOMT, hydroxyindole-*O*-mehyltransferase; MT, melatonin; NAS, *N*-acetylserotonin; TPH, tryptophan hydroxylase.

As one of the major monoaminergic neurotransmitters in the central nervous system, serotonin is involved in various physiological processes, such as the regulation of appetite, aggressiveness, body temperature, fear, mood, pain, reproduction, sleep, and vascular functions [1,2]. Before it becomes a neurotransmitter, serotonin also influences the development and maturation of the mammalian brain [3]. In addition, serotonin acts as a precursor for the synthesis of melatonin, which is mainly produced in the pineal gland of vertebrates. Tryptophan hydroxylase (TPH, EC 1.14.16.4) is the rate-limiting enzyme for serotonin biosynthesis and is the initial activated enzyme in melatonin synthesis. TPH transforms tryptophan (Trp) into 5-hydroxytryptophan (5-HTP) [4], and subsequently, 5-HTP is catalyzed to serotonin (5-hydroxytryptamine, 5-HT) by l-aromatic amino acid decarboxylase (AAAD, EC 4.1.1.28) [5]. Serotonin is further catalyzed by aralkylamine *N*-acetyltransferase (AANAT, EC 2.3.1.87) [6] and acetylserotonin-*O*-methyltransferase (ASMT; EC 2.1.1.4) [7] to finally generate melatonin (MT; see Figure 1).

Tryptophan hydroxylase belongs to the superfamily of aromatic amino acid hydroxylase that includes tyrosine hydroxylase (TH) and phenylalanine hydroxylase (PAH) [8]. Compared with the increasing number of reports concerning the families of TH and PAH [9,10,11,12], studies of TPH appear sparse [13,14], and a comprehensive investigation in vertebrates is unavailable at present. For more than a decade, the TPH family was initially thought to have only one member. However, in 2003, Walther et al. knocked out the *tph* gene (now called *tph1*) in mice. Surprisingly, they discovered that the *tph1*-deficient mice lacked serotonin in the pineal gland and certain peripheral tissues (such as gut and blood). They also observed that the serotonin content in the brainstem of the *tph1*-deficient mice was slightly reduced in comparison to the wild type, suggesting the existence of another *tph* member contributing to the stable concentration of serotonin in the brainstem [13]. Subsequently, they discovered and verified a novel member of the TPH family that they named *tph2*, and thus the classical *tph* gene was renamed *tph1*. It has been shown that *tph1* is mainly distributed in the pineal gland and peripheral gut, spleen, and thymus; *tph2* is predominantly expressed in structures of the central nervous system, such as the brainstem [15]. As summarized in Figure 1, there are two independent serotonin systems in vertebrates that are regulated by two different TPH enzymes. Therefore, these serotonin systems regulated by *tph1* and *tph2* have distinct functions. *tph1* usually plays important roles in peripheral effects such as hemostasis, the immune system, melatonin synthesis, migraine, and vasoconstriction; *tph2* is involved in effects of the central nervous system, such as aggression, anxiety, depression, epilepsy, food intake, migrate and sleep. Only the pathogenesis of migraine may involve both serotonin systems (the overlapped middle part in Figure 1).

Fish are the most diverse vertebrate taxa, and their serotonergic neurons can be identified based on corresponding 5-HT levels. A previous review [16] described *tph* as a specific marker for 5-HT generation. Teleosts experienced an episode of whole genome duplication (WGD) approximately 320 million years ago (Mya) [17,18], resulting in the duplication of certain genes related to 5-HT. After the WGD event, however, these duplicated genes occasionally were lost or became pseudogenes [19]. Hence, more *tph* copies (*tph1a*, *tph1b*, and *tph2*) have been reported in teleost species such as zebrafish, sticklebacks, and medaka [16,20].

Two members (*tph1* and *tph2*) of the TPH family with distinct catalytic activity or substrate specificity had been reported previously [13]. Certain studies already indicated that distinct functions of different family members may have originated from different selection pressures during evolution [21,22]. Investigations of codon substitutions within vertebrate groups also suggested that certain protein families had experienced positive selection, which was considered as an important driving force in protein evolution both from structural and functional views [23,24,25]. To date, although additional copies of *tph* have been reported in fishes, a detailed phylogenetic analysis of these *tph* members among teleosts and tetrapods is lacking, and functional changes related to possible positive selection on different *tph* copies is poorly understood.

In the present study, we focused on 70 vertebrate species with high-quality genome assemblies for extraction of their *tph* family members. Meanwhile, we examined a number of special teleosts that possess some unique living habitats: cavefish (*Sinocyclocheilus anshuiensis*, Sa), amphibious mudskippers (blue-spotted *Boleophthalmus pectinirostris* (BP) and giant-fin *Periophthalmus magnuspinnatus* (PM)), migratory fish (Atlantic salmon (*Salmo salar*), and rainbow trout (*Oncorhynchus mykiss*)). We constructed a comprehensive phylogenetic tree from encoding sequences and implemented synteny analysis among the examined species. Sequence and structural divergences between different TPH members were also compared. Moreover, the biochemical and structural properties of possible positively selected sites in the TPH family were summarized. We attempted to answer the following questions. (1) What are the main structural differences in different members of the TPH family among various taxa of vertebrates? (2) Have some species experienced loss of certain *tph* member(s) during evolution? (3) Did positive selection affect functional changes of the TPH members during evolution?

## 2. Materials and Methods

### 2.1. Sequence Collection

In this study, a total of 70 vertebrate genomes were chosen to extract *tph* genes. Genome sequences of 68 species, including 11 mammals, 18 aves, 9 reptiles, 3 amphibians, and 27 Actinopterygii, with high-quality assemblies, were downloaded from the National Center for Biotechnology Information (NCBI), with the exception of two fish species (the American paddlefish and the Chinese sturgeon), which were assembled based on Illumina and PacBio sequencing using Platanus (version 1.2.4 [26], parameters: −k 31 −s 10 −a 10 −u 0.1 −d 0.4) and DBG2OLC (default version, [27], parameters: k 17 KmerCovTh 6 MinOverlap 80 AdaptiveTh 0.012 RemoveChimera 1) by our lab (Table S1). To further collect the entire length of *tph1* and *tph2* coding sequences in each genome, those reported and verified *tph* genes in human (*Homo sapiens*), chicken (*Gallus gallus*), painted turtle (*Chrysemys picta bellii*), African clawed frog (*Xenopus laevis*), and zebrafish (*Danio rerio*) were downloaded from NCBI as the queries. In general, we selected the sequences from organisms close to the target species as the queries; for most sequences of *tph2* in the amphibians we used human *tph2* (NP_775489.2) as the query. Detailed information concerning the queries is provided in Table S2.

We constructed a local database for each genome and subsequently aligned the sequences through BLAST (version 2.2.28) [28] using the protein-nucleotide aligned strategy with an E-value of 10^−5^. The alignment results were further processed by a Perl script to obtain the best hit of each alignment. Finally, EXONERATE (version 2.2.0) [29] was employed to predict the full length of each *tph* gene.

### 2.2. Sequence Alignment and Phylogenetic Reconstruction

The predicted nucleotide sequences of *tph* genes were initially used for multiple codon-based alignments using the Muscle module in MEGA (version 7.0) [30] and, subsequently, were translated to protein sequences for phylogenetic analyses. ProtTest (version 3.4.2) [31] was used to predict amino acid (aa) substitutions under the Akaike information criterion (AIC). The parameters within the best amino acid substitution of JTT+I+G were input to PhyML (version 3.1) [32] to construct phylogenetic topologies using maximum likelihood (ML) with 1000 bootstrap replicates for the evaluation of their branch supports.

To further confirm the topology yielded by ML, our alignment sequences were also analyzed using Bayesian inference (BI) in MrBayes (version 3.2.6) [33]. We conducted two independent Bayesian searches for 1 × 10^6^ generations with one cold chain and three heated chains per run, and every 100 generations was sampled for each run. The initial 25% of the runs were discarded as a burn-in. A consensus tree was generated with the remaining runs, and Bayesian posterior probabilities were calculated to evaluate the branch supports. The maximum clade credibility tree was identified from the remaining samples using TreeAnnotator (version 1.7.5) [34].

### 2.3. Identification of Conserved Synteny

To examine the conservation of *tph* genes in vertebrates, we searched several genes located upstream and downstream of each *tph* paralog within tetrapods and teleosts through Ensemble datasets. Those genes located upstream and downstream of each *tph* paralog in humans and zebrafish were employed as the reference sequences for searching syntenic locations in the tetrapod and teleost sequences, respectively. Subsequently, we employed the protein aligned to nucleotides strategy to examine these extracted synteny genes in tetrapods and teleosts. Genome assemblies of different species were searched using BLAST with an E-value cutoff at 10^−5^. The best hit was used to identify an occurrence of a collinear gene among the examined species.

### 2.4. Detection of Differences between TPH Protein Sequences

We first selected the two human TPH isoforms to perform the alignment, and then analyzed the differences between TPH1 and TPH2. To further analyze secondary structures of these TPH proteins, we also downloaded the common human TPH1 (5L01) and TPH2 (4V06) protein templates from the Protein Data Bank (PDB) for comparing the differences between TPH1 and TPH2 in vertebrates. One or two representative species from each class were chosen to align with the human TPH1 and TPH2 templates. The examined species included cattle (*Bos taurus*), chicken, zebra finch (*Taeniopygia guttata*), American alligator (*Alligator mississippiensis*), Chinese alligator (*A. sinensis*), tropical clawed frog (*Xenopus tropicalis*), African clawed frog, zebrafish, amphibious teleosts (mudskippers), a *Sinocyclocheilus* cavefish (Sa), and tetraploid *Sinocyclocheilus* fish (Sg and Sr). Previous studies had reported that teleosts such as zebrafish possessed two *tph1* genes (*tph1a* and *tph1b*) [20]; thus, we further chose some representative species from the teleosts to identify the differences between TPH1a and TPH1b. All the alignment results were colorized by TEXshade (version 1.24) [35].

### 2.5. Prediction of Tertiary Structures of TPH Proteins

To understand whether these possible variations could shape the tertiary structures, we predicted the tertiary structure and function of each TPH protein in zebrafish, chicken, and one mudskipper (BP) using SWISS-MODEL [36], an online modeling tool for automated comparison of protein homology. We first uploaded our target sequences and searched for a template that best matched our target sequences in terms of coverage and identity. The best templates of the TPH protein model in human (TPH1: 5L01; TPH2: 4V06) from PDB were downloaded to compare the tertiary structure differences among TPH1a, TPH1b, and TPH2. We subsequently conducted model–template alignment for structural comparisons. Finally, PyMOL (version 2.2) [37] was applied to visualize the predicted TPH protein tertiary structures, and these were adjusted in a similar pattern to identify variations.

### 2.6. Identification of Putative Positively Selected Sites

To evaluate variation in selection pressure between lineages of TPH1 and TPH2, we selected a subset of five species from those shown in Figure S1 to represent the five groups of vertebrates, and we constructed a BI tree for selection pressure analyses. We estimated the ratio of *d_N_/d_S_* (ratio of nonsynonymous substitutions (*d_N_*) to that of synonymous substitutions (*d_S_*)) under two prior assumptions using branch models in the CODEML modules of PAML (version 4) [38]. We first ran a one-ratio model for which one ω value was assumed for all clades. We then ran a two-ratio model for which ω values could change between the TPH1 clade and the TPH2 clade (setting the TPH2 clade as an independently varying target ω2; setting the TPH1 clade as the constant ω1). Likelihood ratio (LR) tests and Chi-square (χ^2^) tests were conducted by comparing the probabilities of the one-ratio and two-ratio models to verify which model best fit the data [39]. Finally, a branch-site model, allowing ω to have more than one change in the target branches, was used to identify which codon(s) had likely undergone positive selection. Those sites with significance (*p*-value less than 0.05) based on the Empirical Bayes method [40] were regarded as positively selected.

## 3. Results

### 3.1. Variation of tph Copy Number in Vertebrates

A total of 184 *tph* nucleotide sequences, including 105 *tph1* (37 *tph1a* and 26 *tph1b* in teleosts, and 42 *tph1* in tetrapods and non-teleosts) and 79 *tph2* were successfully derived from 70 vertebrate species (see Table 1). These sequences and their encoding protein sequences were pooled for further analysis.

**Figure 2:**
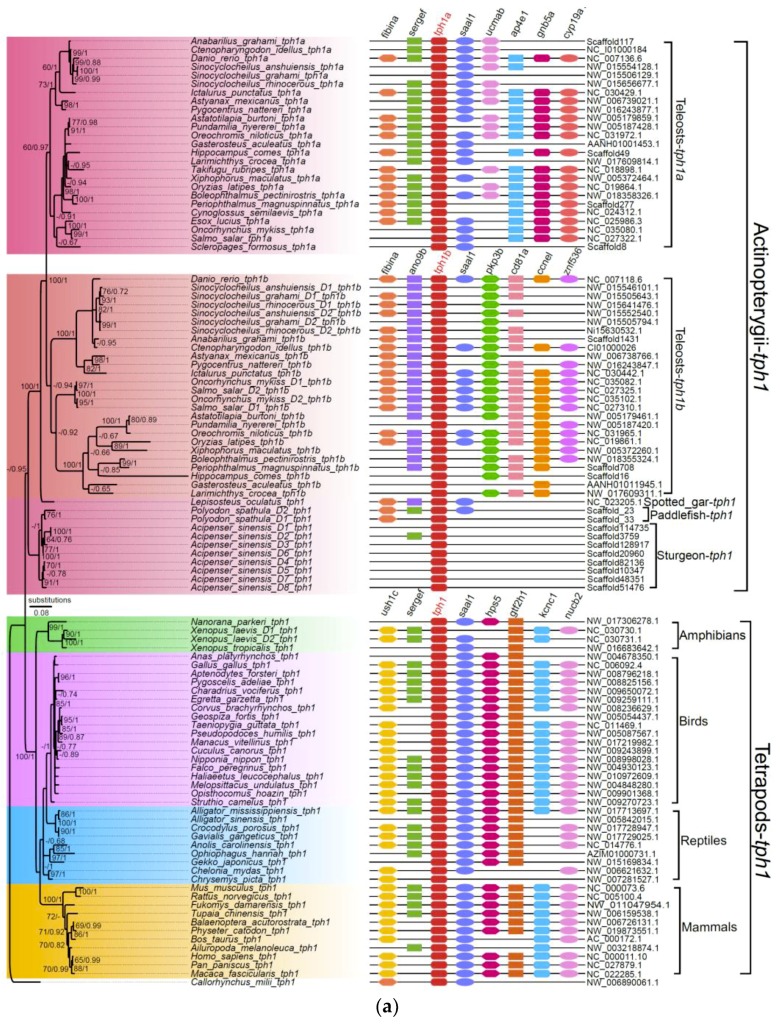

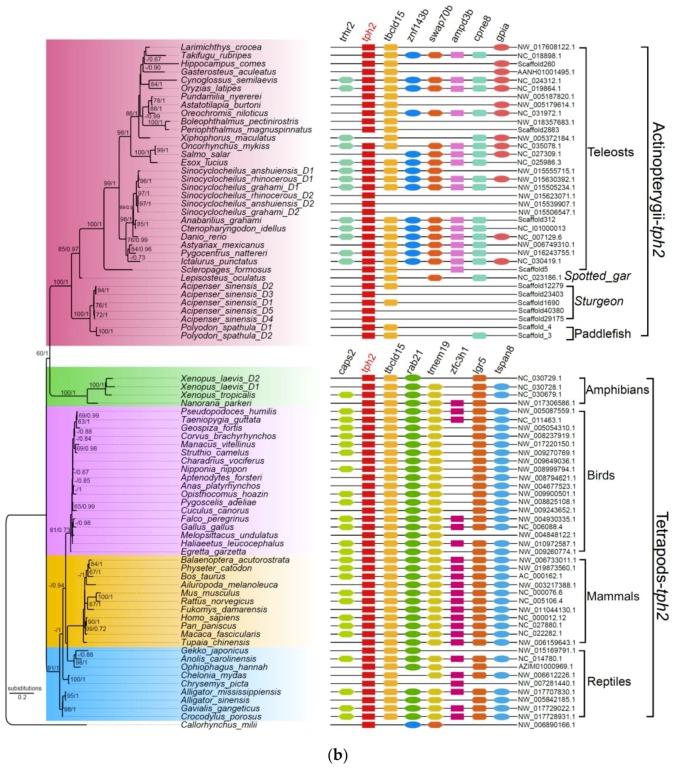
Phylogenetic trees and genome synteny of *tph1* and *tph2* in vertebrates. (**a**) The phylogenetic tree was generated from 105 TPH1 protein sequences (the left part), and the synteny data of *tph1* (the right part) are presented for confirmation. (**b**) The phylogenetic tree was generated from 79 TPH2 protein sequences (the left part), and the synteny data of *tph2* (the right part) are presented for validation. Numbers on the branches from left to right are bootstrap values generated in the PhyML reconstruction and the Bayesian posterior probabilities obtained in the Bayesian inference, respectively. The bootstrap values under 60% and posterior probabilities less than 0.65 are not shown.

In this study, we determined that mammals, birds, reptiles, amphibians, and some basal non-teleost ray-finned fishes, such as the spotted gar, American paddlefish, and Chinese sturgeon, had two *tph* isotypes (*tph1* and *tph2*). For most tetrapods, we observed only one copy of *tph1* and one copy of *tph2*, the one exception being the African clawed frog that had two copies of both *tph1* and *tph2* (see Figure 2 and Table S3). Two copies of *tph1* and *tph2* in the American paddlefish and eight copies of *tph1* and five copies of *tph2* in the Chinese sturgeon were also identified.

Previous studies reported the existence of three *tph* members (defined as *tph1a*, *tph1b*, and *tph2*) in teleosts such as zebrafish, medaka, and sticklebacks [16,20]. In our current work, we further strengthened the finding that most teleosts with the teleost-specific genome duplication (TSGD) presented three *tph* isotypes (*tph1a*, *tph1b*, and *tph2*) with the exception of the Asian arowana (*Scleropages formosus*), fugu (*Takifugu rubripes*), Northern pike (*Esox lucius*), and tongue sole (*Cynoglossus semilaevis*), for which we failed to find *tph1b*. In addition, certain tetraploid fishes that had undergone more than one WGD event, such as the Chinese golden-line fish (Sa, Sr, and Sg), had five *tph* copies (not the expected six); no duplicates of *tph1a* were identified. Other typical tetraploid teleosts such as the rainbow trout and Atlantic salmon had four copies of the *tph* genes, but the duplicated *tph1a* and *tph2* loci were not found (Figure 2 and Table S3).

### 3.2. Phylogenetic Relationships among the tph Genes in Vertebrates

To understand the relationships among these *tph* genes in vertebrates, we constructed a robust phylogenentic tree based on the Bayesian inference (BI) method from the deduced 184 TPH protein sequences. The TPH2 protein sequence from the elephant shark (*Callorhynchus milii*) was employed as the outgroup. According to the well-supported phylogenetic topology (Figure S1), all *tph* genes could be divided into two main groups (*tph1* and *tph2*), and both groups were further divided into two subgroups of tetrapods and Actinopterygii (Figure S1). The tetrapod subgroup was further divided into four main clades (amphibians, mammals, reptiles, and birds).

To avoid overestimation based on the BI method, we also performed a phylogenetic analysis (the left areas of Figure 2a,b) using the maximum likelihood (ML) method on 105 TPH1 and 79 TPH2 protein sequences. We observed that the topologies generated from both ML and BI were similar, indicating a relative stability of the phylogenetic trees. Finally, we employed the ML tree for presentation, and the Bayesian posterior probabilities from the BI analysis were labeled onto each node to indicate the degree of support (Figure 2).

Based on these results, we determined that both tph1 and tph2 were divided into two main clades, one for tetrapods and another for Actinopterygii. We detected in tetrapods and some basal non-teleost ray-finned fishes, namely, the spotted gar, American paddlefish, and Chinese sturgeon, only one isform of tph1. Notably, tph1, in the majority of teleosts that had undergone TSGD, could be further divided into tph1a and tph1b (see more details in the left part of Figure 2a).

### 3.3. Synteny Data

Our synteny data demonstrated that *tph1a*, *tph1b*, and *tph2* share a conserved suite of genes bounding the sequences in both sides, although several genes were not identified in certain species. Genes located upstream and downstream of *tph* between tetrapods and Actinopterygii were different. In general, seven genes (*gtf2h1*, *hps5*, *kcnc1*, *nucb2*, *saal1*, *sergef*, and *ush1c*) were in the neighborhood of the *tph1* gene in tetrapods. Among these seven genes, only two, *saal1* and *sergef*, were identified near the *tph1* gene in Actinopterygii. Similarly, seven genes (*ap4e1*, *cyp19a1*, *fibina*, *gnb5a*, *saal1*, *sergef*, and *ucmab*) were identified near *tph1a*, and seven genes (*ano9b*, *ccne1*, *cd81a*, *fibinb*, *pkp3b*, *saal1*, and *znf536*) were identified near *tph1b* in Actinopterygii. However, only *fibina* and *saal1* were common to the loci neighboring *tph1a* and *tph1b* (see more details in the right part of Figure 2a). The genes located near *tph2* differed between tetrapods and Actinopterygii. Seven genes (*caps2*, *lgr5*, *rab21*, *tbc1d15*, *tmem19*, *tspan8*, and *zfc3h1*) and seven genes (*ampd3b*, *cpne8*, *gpia*, *swap70b*, *tbc1d15*, *trhr2*, and *znf143b*) were located near *tph2* in tetrapods and Actinopterygii, respectively. Only *tbc1d15* was shared between tetrapods and Actinopterygii (see the right part of Figure 2b). In non-teleost ray-finned fishes such as sturgeons and paddlefishes, many genes near to *tph* were not identified in teleosts. However, the genes adjacent to *tph* throughout the tetrapods were identified to a greater degree than in ray-finned fishes based on the results of synteny analyses, indicating that the adjacent regions in tetrapods were more conservative than those in ray-finned fishes (see more details in the right areas of Figure 2).

### 3.4. Sequence Variations and Secondary Structures of the tph Genes

**Figure 3:**
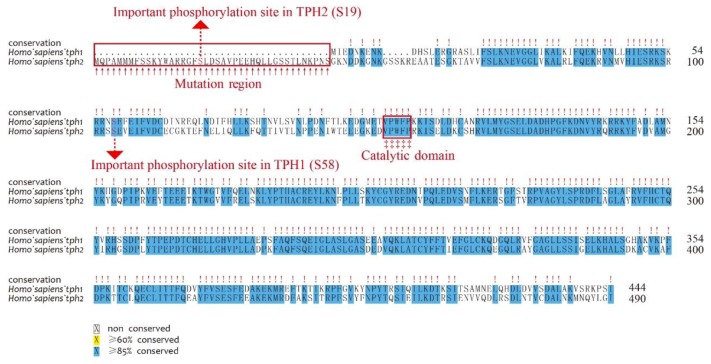
Alignment of human TPH1 and TPH2 protein sequences. Important residues include the mutation region (in the NH2-terminal red box), phosphorylation sites (dashed lines with red arrows), and catalytic domains (‡ with the red box).

We first aligned the protein sequences of human *tph1* and *tph2*, which were located on chromosomes 11 and 12, respectively. Depending on the alignment results, we observed that TPH1 and TPH2 were highly conserved in humans, while TPH2 has an extra length of 41 aa at the NH_2_-terminal (Figure 3). Ser 19 in TPH2 and Ser 58 in TPH1 have been reported as two of the most important phosphorylation sites [13,41]. The TPH enzymes are activated through phosphorylation, and then the phosphorylated TPHs bind to 14-3-3 proteins (a family of acidic, highly homogenous proteins) to increase the stability of these enzymes against degradation [42]. Thus, the absent region in TPH1 in comparison to TPH2 may imply functional divergence despite the existence of other conserved sequence features. The phosphorylation site of Ser 58 in TPH1 was also conserved in TPH2, while TPH2 had an additional phosphorylation site at Ser 19, which may suggest that TPH2 would be more stable and have stronger inhibition of dephosphorylation compared with TPH1. Both TPH1 and TPH2 have a conserved pentapeptide (Val–Pro–Trp–Phe–Pro) catalytic domain that begins at the residue 151 of human TPH2 (see Figure 3).

**Figure 4:**
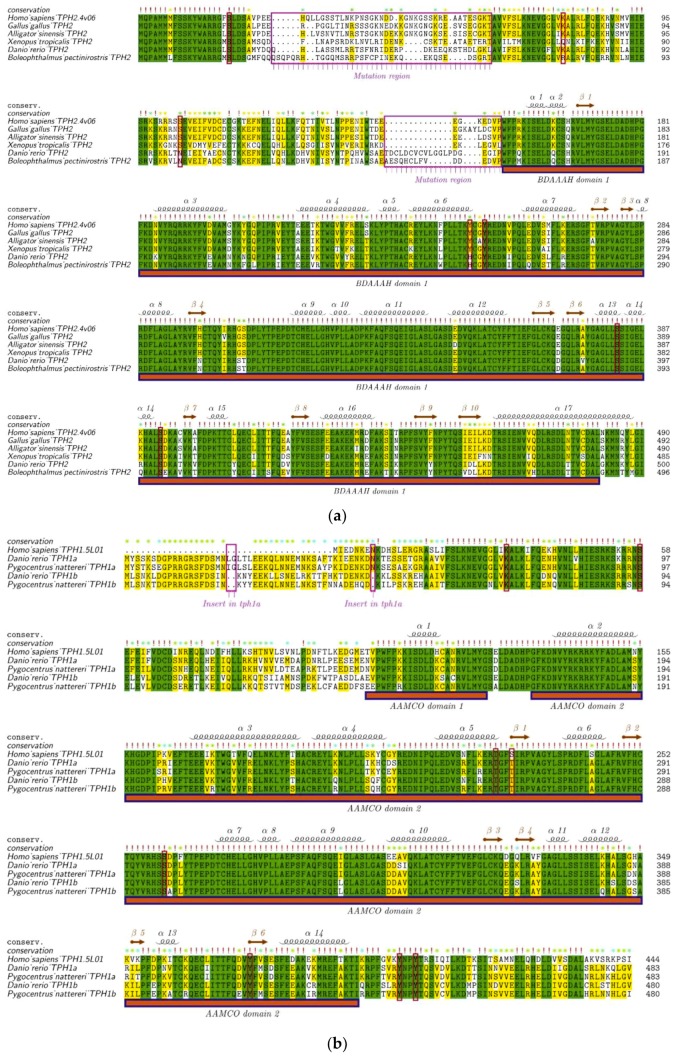

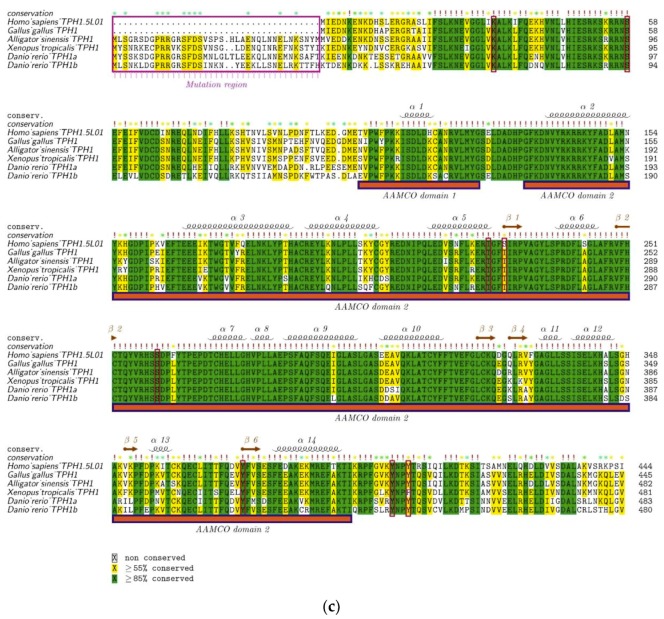
Alignment and secondary structures of TPH protein sequences in vertebrates. (**a**) TPH2 protein sequences from representative vertebrate species were aligned with the human TPH2 and its secondary structure template 4V06. (**b**) Sequence alignment between TPH1a and TPH1b in some representative teleosts is provided for comparison. (**c**) TPH1 protein sequences from representative vertebrates were aligned with the human TPH1 and its secondary structure template 5L01. The mutation regions are marked with rose boxes and upper arrows in each figure. The red boxes denote the phosphorylation sites based on the human templates. Related alignment and secondary structures in more vertebrate species are provided in Figure S2.

To provide estimates for the divergence of TPH1 and TPH2 in vertebrates, we aligned the human structural templates of 5L01 and 4V06 with TPH1 and TPH2 from different species. TPH2 sequences from six representative species in vertebrates were selected to perform the alignment using human TPH2 as the template. We found that variations of TPH2 in the examined vertebrates were mainly present in the NH_2_-terminal and in residues around the first α-helix. The mutation regions of TPH2 among vertebrates are marked with the rose box in Figure 4a. Seven phosphorylation sites (S19, K79, S104, Y249, Y252, S382, and S392) in TPH2, according to the human template, are marked with red boxes in Figure 4a. Among these phosphorylation sites, K79 in humans was changed to Q79 in the amphibians and to R79 in the amphibious mudskippers (BP and PM); S104 in humans was changed to N104 in teleosts; and Y249 in humans was also changed to H249 in teleosts. In general, these TPH2 protein sequences contained one Biopterin_H- and one Biopterin-dependent aromatic amino acid hydroxylase (BDAAAH) domain, and their secondary structures consisted of 17 α-helices and 10 β-strands (Figure 4a). Alignment and secondary structures of TPH2 in additional vertebrate species are provided in Figure S2.

As mentioned above, most teleosts that had undergone TSGD had two isotypes of *tph1* named *tph1a* and *tph1b*. To further detect the differences between *tph1a* and *tph1b*, we selected two representative species from the teleosts to perform the alignment with the human TPH1 (protein ID: 5L01) as the template. TPH1a contains 483 aa, while TPH1b contains 480 aa. TPH1a has two aa (LG or IG) inserted at sites 20 and 21 and one aa of N inserted at site 47 compared to TPH1b. These three insertion sites in TPH1a are marked with rose boxes in Figure 4b. Many variable sites between TPH1a and TPH1b were also identified near the first α-helix. However, the phosphorylation sites (marked within red boxes) are almost identical between TPH1a and TPH1b. In general, the domain regions were highly conserved between TPH1a and TPH1b in teleosts (Figure 4b). Alignment and secondary structures of TPH1a and TPH1b in additional teleost species are presented in Figure S2.

Similarly, alignment of TPH1 proteins in five representative species of vertebrates is shown in Figure 4c. The alignment indicated that fishes, amphibians, and reptiles had about 39 more aa compared with mammals and birds at the NH2-terminal. Eight phosphorylation sites (K33, S58, T205, S228, S260, Y373, Y401, and Y404 in TPH1) are marked with red boxes in Figure 4c. Among these phosphorylation sites, only S288 in humans was changed to T228 in other tetrapods and fishes, and Y404 in human was changed to F404 in amphibians. Generally, TPH1 contains two aromatic amino acid monoxygenases, catalytic and oligomerization (AAMCO) domains, and the secondary structures of TPH1 consist of 14 α-helices and six β-strands (see Figure 4c). Alignment and secondary structures of TPH1 in additional teleost species are shown in Figure S2.

### 3.5. Predicted Three-Dimensional (3D) Structures of the TPH Proteins

**Figure 5:**
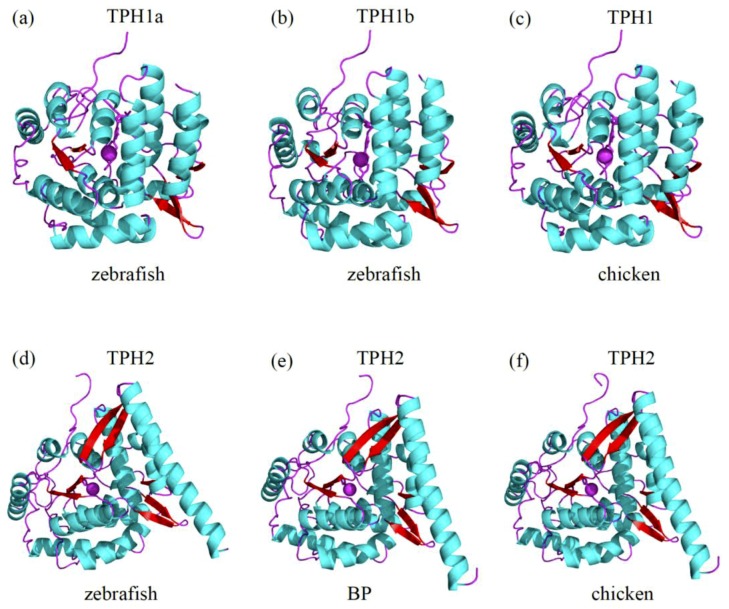
Predicted 3D structures of representative TPH proteins. Comparisons of the 3D structures of zebrafish TPH1a (**a**), zebrafish TPH1b (**b**), chicken TPH1 (**c**), zebrafish TPH2 (**d**), BP TPH2 (**e**), and chicken TPH2 (**f**) are illuminated in Section 3.5. Helices of the catalytic domains and β-strands are colored sky blue and red, respectively. Loop regions are marked in purple.

In comparing the three-dimensional (3D) structure of TPH1 between zebrafish and chickens, we observed that both shared the same conformation of the structural elements (α-helices and β-strands), while some slight structural modifications were present in the loops (Figure 5a,c). The 3D structures of TPH1a and TPH1b in zebrafish were also strikingly similar, except for minor differences in the loop regions (Figure 5a,b).

Similarly, we compared the 3D structures of TPH2 among zebrafish, amphibious mudskippers (BP), and chickens. The structural elements of α-helices and β-strands also shared the same conformation, except for some slight variation in the loops (Figure 5d–f).

It seems that the loop regions in these TPH proteins were relatively flexible. However, the 3D structures of TPH1 and TPH2 showed obvious differences, and the divergences were located not only in the structural elements of α-helices and β-strands but also in the loops (see Figure 5), which was consistent with the functional differences between *tph1* and *tph2*.

### 3.6. Detection of Putative Positively Selected Sites in the TPH Family

Researchers had reported that *tph1* from peripheral tissues and *tph2* from the brainstem demonstrated biochemical and functional differences [13,41]. In our present study, we also verified that TPH1 and TPH2 had structural differences based on our aforementioned prediction of 3D structures. However, positive selection has been identified as an important driving force in protein evolution of both structure and function based on phylogenetic analyses [43]. We therefore characterized the selection pressures of different members in the TPH family.

**Figure 6:**
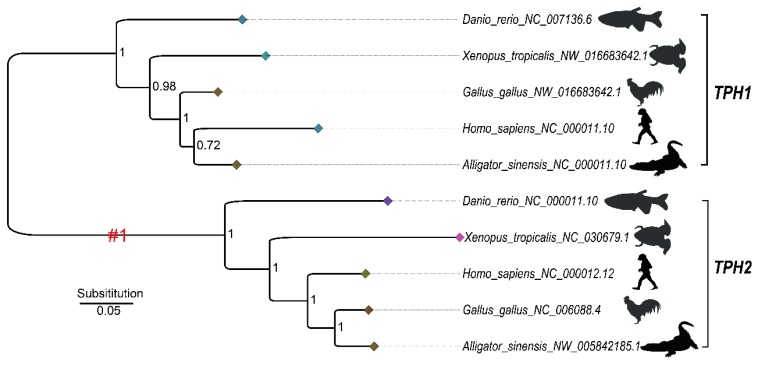
The Bayesian inference (BI) tree based on five TPH1 and TPH2 proteins selected from those shown in Figure S1 to represent the five major groups of vertebrates for selection pressure analysis. #1 denotes the foreground of the TPH2 clade. Numbers in the topology indicate the Bayesian posterior probabilities. The diamonds at the tips of each branch are colored based on the branch length, with a darker color representing a shorter evolutionary branch.

A phylogenetic tree for selection pressure analysis was constructed based on a subset of five TPH1 and five TPH2 protein sequences that were selected from those shown in Figure S1 to represent the five major groups of vertebrates (Figure 6). We employed a one-ratio or two-ratio branch model in PAML to check for significant differences in selection pressure between the clades of TPH1 and TPH2 (Figure 6). The log-likelihood values under one-ratio and two-ratio models were ln L = −8188.872713 and ln L = −8182.688839, respectively. Our likelihood ratio tests suggested rejection of the one-ratio model (*p* < 0.001); the branch model test suggested that selection pressure significantly differed between the clades of TPH1 and TPH2. We therefore concluded that selection pressure varied between the clades of TPH1 and TPH2.

**Figure 7:**
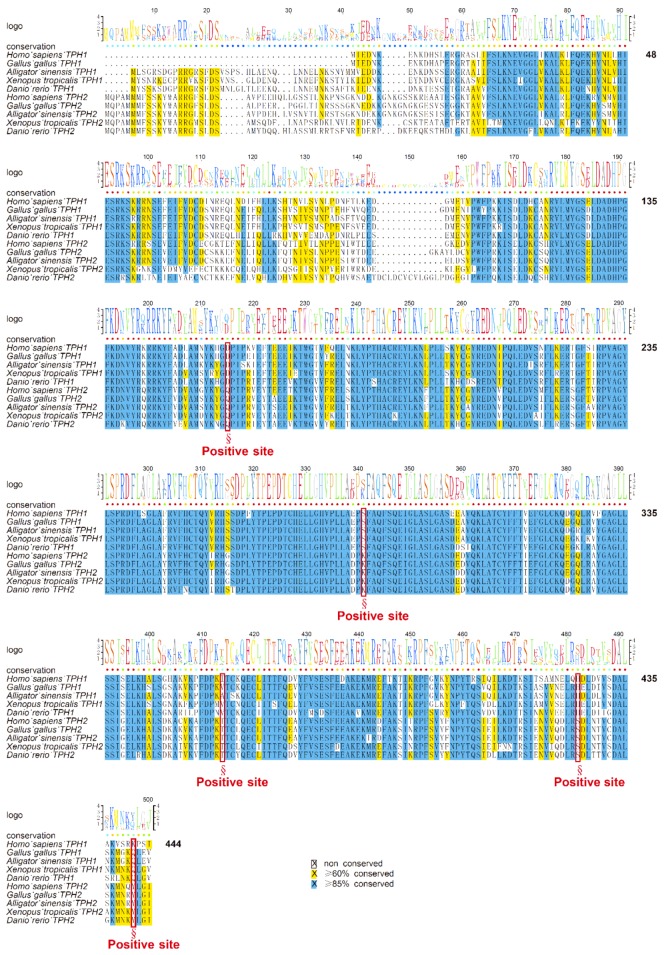
Alignment of the datasets for selection pressure analysis. The five positively selected sites predicted by the branch-site model test are marked with red boxes and emphasized with section marks (§).

The mean ω (d_N_/d_S_) values for the TPH1 and TPH2 clades were 0.047 and 4.592, respectively, indicating that the TPH2 clade may have experienced positive selection. Furthermore, we estimated that some codons had undergone positive selection. Based on Empirical Bayes analysis, we further identified five positively selected candidate sites (D159, S285, I358, H426, and K441; see Figure 7), and the posterior probabilities of these sites were greater than 0.99. The positively selected site D159 was near to the pentapeptide (Val–Pro–Trp–Phe–Pro) catalytic active domain, while other positive sites were next to the phosphorylation site region or α-helix and loops of the TPH family (see Figure 7).

We also added ten additional vertebrate sequences, with five belonging to TPH1 and five belonging to TPH2 (see Figure S3) to form a more comprehensive dataset to confirm the analysis of positive selection. The results also supported the hypothesis that the branch of TPH2 possibly experienced positive selection in comparison to TPH1 (the log-likelihood values under one-ratio and two-ratio models were ln L = −11719.144671 and ln L = −11713.763977, respectively, and the *p*-value from the χ^2^ test was less than 0.001). We performed the analysis again under branch site models and determined that the list of inferred positively selected sites was the same as in the previous version.

## 4. Discussion

### 4.1. Possible Reasons for Variation of tph Copy Number in Vertebrates

Whole genome duplication has been proposed to provide additional genetic material for the appearance of new genes, allowing organisms to acquire novel phenotypes to survive natural challenges [44]. In vertebrates, two rounds of WGD occurred in their common ancestor [45,46]. Specifically, the first round happened before the split between Gnathostomes and Agnatha (jawless vertebrates), and the second round occurred before the split between Chondrichthyes and Osteichthyes [47,48]. Furthermore, the teleost lineage had undergone an additional WGD known as the TSGD approximately 320 Mya [17,18]. Besides the TSGD at the base of the teleost lineage, recent genome duplication events had occurred within some teleost lineages, including the salmonid-specific genome duplication 80 Mya [49,50], and the common carp lineage appeared approximately 8 Mya [51]. In addition to teleosts, some basal ray-finned fish lineages, such as sturgeons and paddlefishes in Acipenserifomes, also had experienced tetraploidization events around 184 Mya [52].

According to our results, most tetrapods examined in this study and some basal non-teleost ray-finned fishes (e.g., the spotted gar) had single copies of *tph1* and *tph2*, with the exception of *Xenopus laevis*, which had two copies of both *tph1* and *tph2*. It has been reported that *X. laevis* had undergone recent tetraploidization approximately 40 Mya [53], and thus the doubled copies of *tph1* and *tph2* were generated in this frog species. This WGD event in *X. laevis* might have been propitious for adapting to various environmental factors such as salt, drought, cold, and disease compared to the closely-related diploid species such as *X. tropicalis*. Spotted gar diverged from the teleost lineage before the TSGD and thus was not affected by the TSGD event. Furthermore, Acipenserifomes experienced their own lineage-specific polyploidization event; thus we detected two copies of *tph1* and *tph2* in the American paddlefish and eight copies of *tph1* and five copies of *tph2* in the Chinese sturgeon.

Researchers had reported that zebrafish, medaka, and sticklebacks had three *tph* isotypes named *tph1a*, *tph1b*, and *tph2* [16,20]. Interestingly, based on a comparative genome-wide survey, we found that most teleosts with the TSGD had three *tph* isotypes, except for Asian arowana, fugu, Northern pike, and tongue sole, which had lost the *tph1b* gene. For the common teleosts, all three *tph* isotypes were present in single copies. However, tetraploid fishes such as the golden-line fishes (Sa, Sr, and Sg) had five copies (not the expected six) of the three *tph* isotypes, including three copies of *tph1* and two copies of *tph2* (with the possible loss of one *tph1a*). Other typical tetraploid teleosts such as the rainbow trout and Atlantic salmon, which had undergone the salmonid-specific genome duplication, had four copies of *tph* genes, but copies of duplicated *tph1a* and *tph2* were not found. The most frequent fate of duplicated genes after WGD is nonfunctionalization; therefore, these duplicated genes often experienced gene loss [54]. We propose that the main cause of copy number variation of *tph* in vertebrates was a combination of WGD and gene loss, as in the cases of *aaad* [55], *aanat* [56], and *asmt* [57], which encode the remaining three enzymes for melatonin biosynthesis (Figure 1). Moreover, incomplete annotation of some genomes may be one of the reasons for the lack of identification of certain genes in some species. Copy numbers between diploid and tetraploid species do not always correspond to a 1:2 ration because of selective loss of certain copies. In fact, this phenomenon seems to be particularly common in teleosts.

Moreover, our synteny analysis revealed that almost all *tph1* and *tph2* genes were localized on different scaffolds in the same species, except for fugu, Northern pike, and tongue sole. Interestingly, we found that the synteny genes were not conserved between tetrapods and Actinopterygii. Only two genes, *saal1* and *sergef*, near the *tph1* gene, were shared in Actinopterygii and tetrapods. Furthermore, synteny genes located near *tph1a* and *tph1b* in the teleosts were not completely consistent. Only *tbc1d15* was shared by tetrapods and Actinopterygii among genes localized near the downstream and upstream areas of *tph2*. It seems that the *tph* gene family experienced rearrangement during the evolution from teleosts to tetrapods. Meanwhile, we also observed that the synteny of *tph* in tetrapods was generally greater than that of teleosts. This phenomenon may be due to the fact that teleost lineages are more prone to interchromosomal rearrangements than tetrapods, resulting in shorter conserved syntenic blocks in teleosts compared with tetrapods [58,59]. However, the TSGD events had also led to shorter syntenic blocks through differential gene loss without rearrangements. Differential loss of blocks of duplicated genes can cause synteny disruption, contributing to shorter syntenic blocks in teleosts than in non-teleost vertebrates [54]. The disrupted syntenic blocks are very common in teleost genomes. For example, Ravi et al. (2013) reported that the syntenic block of the *Pax6* locus contained six genes, and this block was completely conserved in tetrapods. However, in zebrafish, *Pax6* was duplicated to *pax6a* and *pax6b*, owing to the TSGD, and the blocks of *pax6a* and *pax6b* retained three and four genes, respectively [60].

### 4.2. Adaptive Evolution of TPHs in Vertebrates and Impact on Human Health

Elucidating the mechanisms of protein functional diversity and understanding how enzyme families evolve are core issues in evolutionary biology. Catalytic activity toward various substrates and substrate specificity are the key functional properties of an enzymatic protein. Enzyme families with divergent functions usually acquire specific activities relating to structural and functional variations caused by amino acid substitutions during evolution [61]. Numerous phylogenetic analyses based on amino acid substitutions have suggested that many enzymatic protein families have undergone positive selection [24,62], which has been considered as an important driving force in protein evolution.

The TPH family has tissue-specific expression patterns: *tph1* is distributed in the pineal gland and peripheral tissues (gut, spleen, and thymus), while *tph2* is predominantly expressed in the central tissue of the brainstem [15]. Thus, there are two serotonin systems in vertebrates independently regulating distinct functions (Figure 1). However, the lack of comprehensive structural and functional analyses has generally hindered attempts to elaborate this pattern. Structural and functional characteristics of proteins are defined into rigid and flexible regions. Rigidity plays an important role in the integrity of protein folding, while flexibility is important for binding and catalyzing different substrates [63]. In the present study, we compared the 3D structures of TPHs in representative species of vertebrates. The 3D structures of TPH1 and TPH2 indicated that the structural elements of α-helices and β-strands generally shared the same conformation, but some slight structural modifications were present in the loops. However, when we compared the 3D structures of TPH1 and TPH2, we observed obvious differences. The divergence was not only in the loops but also in the structural elements of α-helices and β-strands. These divergences are consistent with the differences in function between *tph1* and *tph2*.

Positive selection acts as an important driving force in protein evolution in both structure and function. Estimating the ratio of *d_N_* to that of *d_S_* between homologous protein coding genes is a significant indictor of positive selection at the molecular level. Ratios of *d_N_/d_S_* (ω) in lineages that have experienced selection may be different from those of other lineages. Previous studies reported evolutionary divergence from both structural and functional views in the TPH family [8,21]. Thus, we further analyzed the selective aspects of the functional and structural divergence of the TPH family and attempted to elucidate patterns of positively selected sites. We detected five positively selected sites in TPH2. Conventional theory emphasizes that replacements in active sites of amino acids will lead to changes in a protein’s function. TPH1 and TPH2 have a conserved pentapeptide (Val–Pro–Trp–Phe–Pro) catalytic active domain in humans (see Section 2.4). The five positively selected sites detected in our present study, however, were not in the catalytic domain. As we reported, the positively selected site of D159 was near the pentapeptide catalytic domain, while other positively selected sites were near the phosphorylation sites or the structural elements (α-helix and loops) of TPHs. Our present study suggests that mutations outside the active regions may have effects on the proteins, despite that mutations close to the active sites were apparently more effective than those more distant. This phenomenon resembles the pine glutathione S-transferase (GST) enzyme family investigated by Lan et al. (2013), in which three of the five positively selected sites detected in their study were located in the nonactive sites [43]. Similarly, positively selected sites were also detected outside the active region of the JGW family in Drosophila, and this generated a new dehydrogenase with varied substrate specificity compared to the ancestral protein [64]. The positive selection acting on residues adjacent to the active sites, rather than direct action on the active sites, may be a general mechanism for the functional diversification of the enzyme families [43,64]. Meanwhile, protein loop regions and α-helix regions are relatively flexible compared with β-strands. In our present study, all five positively selected sites in the TPH family were in the loops and α-helix, suggesting that the TPH proteins could tolerate structural modification. In summary, the positively selected sites detected in the TPH family may affect TPH activity and substrate specificity.

In addition, recent advances have implicated serotonin as a regulator of inflammation [65], proliferation [66], regeneration [67], and repair [68]. Interestingly, a recent population-based study showed that a high intake of selective serotonin reuptake inhibitors was correlated with a reduced incidence of colorectal cancer, suggesting a biological role of serotonin in colorectal cancer growth in vivo [69]. Furthermore, the role of serotonin in tumor biology in vivo has been elucidated using a genetic model of serotonin deficiency (*tph1*-/-) in mice, and researchers concluded that serotonin regulated angiogenesis in colorectal cancer allografts by influencing matrix metalloproteinase 12 expression in tumor-infiltrating macrophages, thereby affecting the production of circulating angiostatin [70]. Therefore, serotonin-related pathways may represent a new therapeutic target for cancer treatment. Our current study provides basic information necessary for further study of serotonin’s role in human health.

## 5. Conclusions

Our present study is the first comprehensive report concerning *tph* evolution in vertebrates. We investigated many features of the *tph* family to provide novel insights into the evolution of *tph* genes from a genomic view. Through genome-wide alignment, we found that tetrapods and non-teleosts had two *tph* isotypes (*tph1* and *tph2*); however, in the teleost lineage, *tph1* had further diverged into *tph1a* and *tph1b*. Therefore, most of the teleosts that had undergone the TSGD event had three *tph* isotypes: *tph1a*, *tph1b*, and *tph2*. Copy numbers between diploid and tetraploid species did not always correspond to a 1:2 ratio, and we estimated that the WGD and gene loss generated the corresponding variation in *tph* gene numbers in vertebrates. Several important sites of TPH proteins were analyzed for structural comparison, and differences of TPHs in vertebrates were mainly focused at the NH_2_-terminal. Tertiary structures of TPH1 and TPH2 showed obvious differences in the structural elements (α-helix and β-strands) and loops. Five positively selected sites were characterized in the TPH family, and these sites may significantly affect the enzyme activity and substrate specificity. This result was consistent with tissue-specific transcription patterns of *tph1* and *tph2* and differential roles in the serotonin systems of vertebrates. In summary, through these analyses and comparisons, we provided novel insights into the molecular evolution of the TPH family in vertebrates.

## Figures and Tables

**Table 1:** Distribution of the tryptophan hydroxylase (TPH) family in the selected vertebrate genomes.

Class	Species Number	*tph1 (tph1a)*	*tph1b*	*tph2*
Mammals	11	11	0	11
Birds	18	18	0	18
Reptiles	9	9	0	9
Amphibians	3	4	0	4
Actinopterygii	29	37	26	37
Total	70	79	26	79

## Supplementary Materials

Supplementary materials can be found online at https://www.mdpi.com/2073-4425/10/3/203/s1. Table S1. GenBank ID of the selected 70 vertebrate genomes. Table S2. Queries used for extraction of *tph* genes from the examined 70 vertebrate species. Table S3. Copy number variation in *tph* genes in the selected vertebrate genomes. Figure S1. BI tree deduced from the 184 vertebrate TPH protein sequences. Figure S2. Alignment and secondary structures of TPH protein sequences in additional vertebrate species. Figure S3. The BI tree based on TPH1 and TPH2 proteins in additional vertebrate species for selection pressure analyses.

## Abbreviations

*ampd3b*: Adenosine Monophosphate Deaminase 3; *ano9b*, anoctamin-9-like; *ap4e1*, Adaptor Related Protein Complex 4 Subunit Epsilon 1; *caps2*, Calcyphosine 2; *ccne1*, Cyclin E1; *cd81a*, CD81 antigen; *cpne8*, Copine 8; *cyp19a1*, Cytochrome P450 Family 19 Subfamily A Member 1; *d_S_*/*d_N_*, ratio of nonsynonymous substitutions (*d_N_*) to that of synonymous substitutions (*d_S_*); *fibina*, Fin Bud Initiation Factor Homolog; *gnb5a*, G Protein Subunit β 5 A; *gpia*, Platelet membrane glycoprotein Ia; *gtf2h1*, General Transcription Factor IIH Subunit 1; *hps5*, Biogenesis of Lysosomal Organelles Complex 2 Subunit 2; *kcnc1*, Potassium Voltage-Gated Channel Subfamily C Member 1; *lgr5*, Leucine Rich Repeat Containing G Protein-Coupled Receptor 5; *nucb2*, Nucleobindin 2; *pkp3b*, Plakophilin 3; *rab21*, RAB21, Member RAS Oncogene Family; *saal1*, Serum Amyloid A Like 1; *sergef*, Secretion Regulating Guanine Nucleotide Exchange Factor; *swap70b*, Switching B Cell Complex Subunit SWAP70; *tbc1d15*, TBC1 Domain Family Member 15; *tmem19*, Transmembrane Protein 19; *trhr2*, Thyrotropin Releasing Hormone Receptor 2; TSGD, teleost-specific genome duplication; *tspan8*, Tetraspanin 8; *ucmab*, Upper Zone of Growth Plate and Cartilage Matrix Associated B; *ush1c*, USH1 Protein Network Component Harmonin; WGD, whole genome duplication; *zfc3h1*, Zinc Finger C3H1-Type Containing; *znf143b*, Zinc Finger Protein 143b; *znf536*, Zinc Finger Protein 536.
