# Supplementary material for: Molecular Evolution of Tryptophan Hydroxylases in Vertebrates: A Comparative Genomic Survey
Source: Genes (Basel). 2019 Mar 8;10(3):203. doi: 10.3390/genes10030203 (PMC6470480; doi:10.3390/genes10030203)
Supplement: Supplementary file 1 [file genes-10-00203-s001.zip › Supplementary_Materials/Figure S1.pdf]

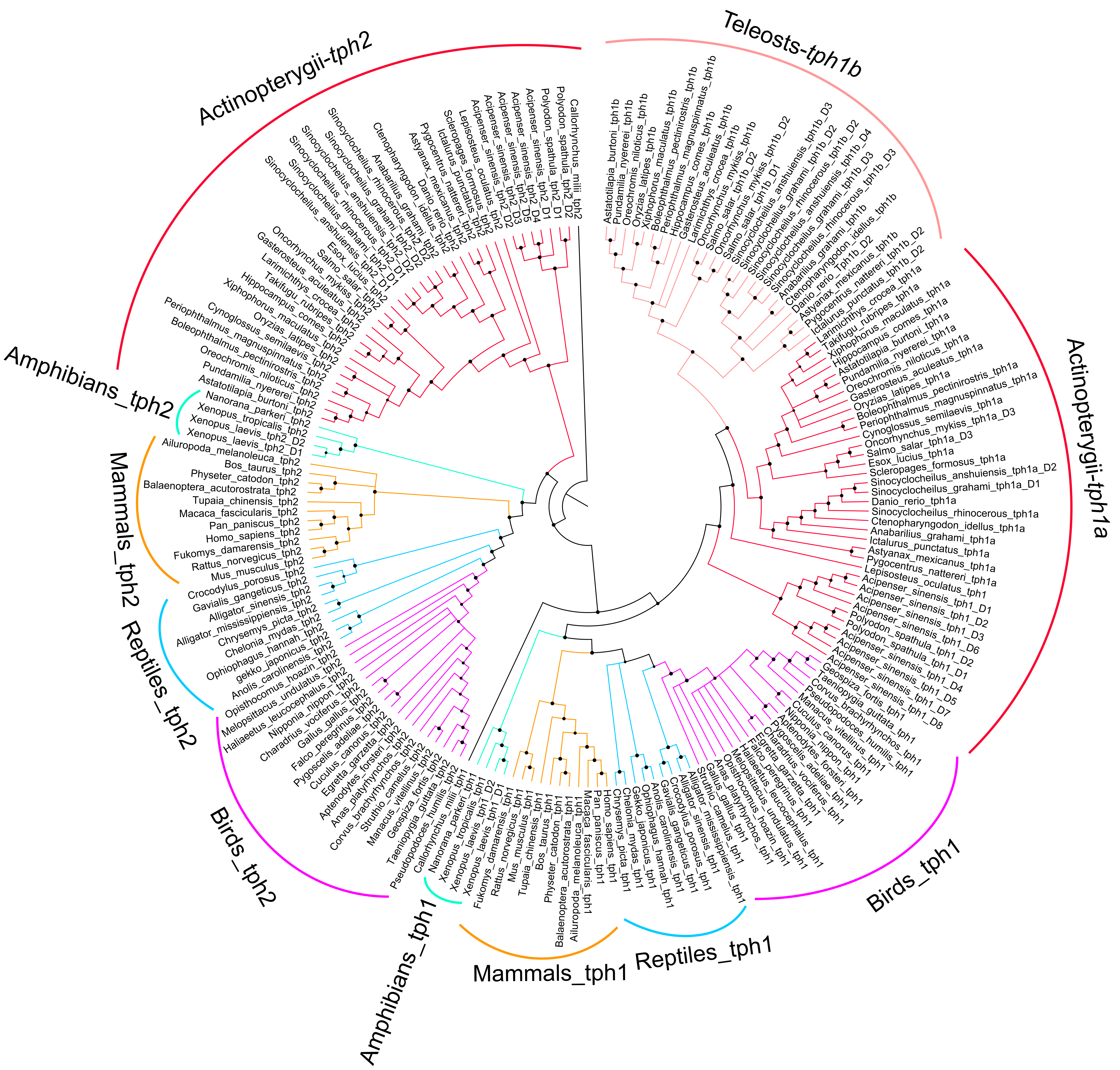

Teleosts-tph1b
Astatotilapia_burtoni_tph1b
Pundamilia_nyererei_tph1b
Oreochromis_niloticus_tph1b
Oryzias_latipes_tph1b
Xiphophorus_maculatus_tph1b
Boleophthalmus_pectinirostris_tph1b
Periophthalmus_magnuspinnatus_tph1b
Hippocampus_comes_tph1b
Gasterosteus_aculeatus_tph1b
Larimichthys_crocea_tph1b
Oncorhynchus_mykiss_tph1b_D2
Salmo_salar_tph1b
Oncorhynchus_mykiss_tph1b_D1
Salmo_salar_tph1b_D2
Sinocyclocheilus_anshuiensis_tph1b_D3
Sinocyclocheilus_grahami_tph1b_D2
Sinocyclocheilus_rhinocerous_tph1b_D2
Sinocyclocheilus_anshuiensis_tph1b_D4
Sinocyclocheilus_grahami_tph1b_D3
Sinocyclocheilus_rhinocerous_tph1b_D3
Anabarilius_grahami_tph1b
Ctenopharyngodon_idellus_tph1b
Danio_rerio_Tph1b_D2
Astyanax_mexicanus_tph1b
Pygocentrus_nattereri_tph1b_D2
Ictalurus_punctatus_tph1b_D2
Actinopterygii-tph1a
Larimichthys_crocea_tph1a
Takifugu_rubripes_tph1a
Xiphophorus_maculatus_tph1a
Hippocampus_comes_tph1a
Astatotilapia_burtoni_tph1a
Pundamilia_nyererei_tph1a
Oreochromis_niloticus_tph1a
Gasterosteus_aculeatus_tph1a
Oryzias_latipes_tph1a
Boleophthalmus_pectinirostris_tph1a
Periophthalmus_magnuspinnatus_tph1a
Cynoglossus_semilaevis_tph1a
Oncorhynchus_mykiss_tph1a_D3
Salmo_salar_tph1a_D3
Esox_lucius_tph1a
Scleropages_formosus_tph1a
Sinocyclocheilus_anshuiensis_tph1a_D2
Sinocyclocheilus_grahami_tph1a_D1
Danio_rerio_tph1a
Sinocyclocheilus_rhinocerous_tph1a
Ctenopharyngodon_idellus_tph1a
Anabarilius_grahami_tph1a
Ictalurus_punctatus_tph1a
Astyanax_mexicanus_tph1a
Pygocentrus_nattereri_tph1a
Lepisosteus_oculatus_tph1
Acipenser_sinensis_tph1_D1
Acipenser_sinensis_tph1_D2
Acipenser_sinensis_tph1_D6
Acipenser_sinensis_tph1_D3
Polyodon_spathula_tph1_D2
Polyodon_spathula_tph1_D1
Acipenser_sinensis_tph1_D4
Acipenser_sinensis_tph1_D5
Acipenser_sinensis_tph1_D7
Acipenser_sinensis_tph1_D8
Birds_tph1
Geospiza_fortis_tph1
Taeniopygia_guttata_tph1
Corvus_brachyrhynchos_tph1
Pseudopodoces_humilis_tph1
Manacus_vitellinus_tph1
Cuculus_canorus_tph1
Nipponia_nippon_tph1
Aptenodytes_forsteri_tph1
Pygoscelis_adeliae_tph1
Charadrius_vociferus_tph1
Egretta_garzetta_tph1
Falco_peregrinus_tph1
Haliaeetus_leucocephalus_tph1
Melopsittacus_undulatus_tph1
Opisthocomus_hoazin_tph1
Anas_platyrhynchos_tph1
Gallus_gallus_tph1
Struthio_camelus_tph1
Reptiles_tph1
Alligator_mississippiensis_tph1
Alligator_sinensis_tph1
Crocodylus_porosus_tph1
Gavialis_gangeticus_tph1
Anolis_carolinensis_tph1
Gekko_japonicus_tph1
Ophiophagus_hannah_tph1
Chelonia_mydas_tph1
Chrysemys_picta_tph1
Mammals_tph1
Homo_sapiens_tph1
Pan_paniscus_tph1
Macaca_fascicularis_tph1
Ailuropoda_melanoleuca_tph1
Balaenoptera_acutorostrata_tph1
Physeter_catodon_tph1
Bos_taurus_tph1
Tupaia_chinensis_tph1
Mus_musculus_tph1
Rattus_norvegicus_tph1
Fukomys_damarensis_tph1
Amphibians_tph1
Xenopus_laevis_tph1_D1
Xenopus_laevis_tph1_D2
Xenopus_tropicalis_tph1
Nanorana_parkeri_tph1
Callorhynchus_milii_tph1
Birds_tph2
Pseudopodoces_humilis_tph2
Taeniopygia_guttata_tph2
Geospiza_fortis_tph2
Manacus_vitellinus_tph2
Struthio_camelus_tph2
Corvus_brachyrhynchos_tph2
Anas_platyrhynchos_tph2
Aptenodytes_forsteri_tph2
Egretta_garzetta_tph2
Cuculus_canorus_tph2
Pygoscelis_adeliae_tph2
Falco_peregrinus_tph2
Gallus_gallus_tph2
Charadrius_vociferus_tph2
Nipponia_nippon_tph2
Haliaeetus_leucocephalus_tph2
Melopsittacus_undulatus_tph2
Opisthocomus_hoazin_tph2
Reptiles_tph2
Anolis_carolinensis_tph2
gekko_japonicus_tph2
Ophiophagus_hannah_tph2
Chelonia_mydas_tph2
Chrysemys_picta_tph2
Alligator_mississippiensis_tph2
Alligator_sinensis_tph2
Gavialis_gangeticus_tph2
Crocodylus_porosus_tph2
Mammals_tph2
Mus_musculus_tph2
Rattus_norvegicus_tph2
Fukomys_damarensis_tph2
Homo_sapiens_tph2
Pan_paniscus_tph2
Macaca_fascicularis_tph2
Tupaia_chinensis_tph2
Balaenoptera_acutorostrata_tph2
Physeter_catodon_tph2
Bos_taurus_tph2
Ailuropoda_melanoleuca_tph2
Amphibians_tph2
Xenopus_laevis_tph2_D1
Xenopus_laevis_tph2_D2
Xenopus_tropicalis_tph2
Nanorana_parkeri_tph2
Actinopterygii-tph2
Astatotilapia_burtoni_tph2
Pundamilia_nyererei_tph2
Oreochromis_niloticus_tph2
Boleophthalmus_pectinirostris_tph2
Periophthalmus_magnuspinnatus_tph2
Cynoglossus_semilaevis_tph2
Oryzias_latipes_tph2
Xiphophorus_maculatus_tph2
Hippocampus_comes_tph2
Takifugu_rubripes_tph2
Larimichthys_crocea_tph2
Gasterosteus_aculeatus_tph2
Oncorhynchus_mykiss_tph2
Salmo_salar_tph2
Esox_lucius_tph2
Sinocyclocheilus_anshuiensis_tph2_D1
Sinocyclocheilus_grahami_tph2_D1
Sinocyclocheilus_rhinocerous_tph2_D1
Sinocyclocheilus_anshuiensis_tph2_D2
Sinocyclocheilus_grahami_tph2_D2
Sinocyclocheilus_rhinocerous_tph2_D2
Anabarilius_grahami_tph2
Ctenopharyngodon_idellus_tph2
Danio_rerio_tph2
Astyanax_mexicanus_tph2
Pygocentrus_nattereri_tph2
Ictalurus_punctatus_tph2
Scleropages_formosus_tph2
Lepisosteus_oculatus_tph2
Acipenser_sinensis_tph2_D2
Acipenser_sinensis_tph2_D3
Acipenser_sinensis_tph2_D5
Acipenser_sinensis_tph2_D4
Acipenser_sinensis_tph2_D1
Polyodon_spathula_tph2_D1
Polyodon_spathula_tph2_D2
Callorhynchus_milii_tph2
