# Supplementary material for: Molecular Evolution of Tryptophan Hydroxylases in Vertebrates: A Comparative Genomic Survey
Source: Genes (Basel). 2019 Mar 8;10(3):203. doi: 10.3390/genes10030203 (PMC6470480; doi:10.3390/genes10030203)
Supplement: Supplementary file 1 [file genes-10-00203-s001.zip › Supplementary_Materials/Figure S2-a.pdf]

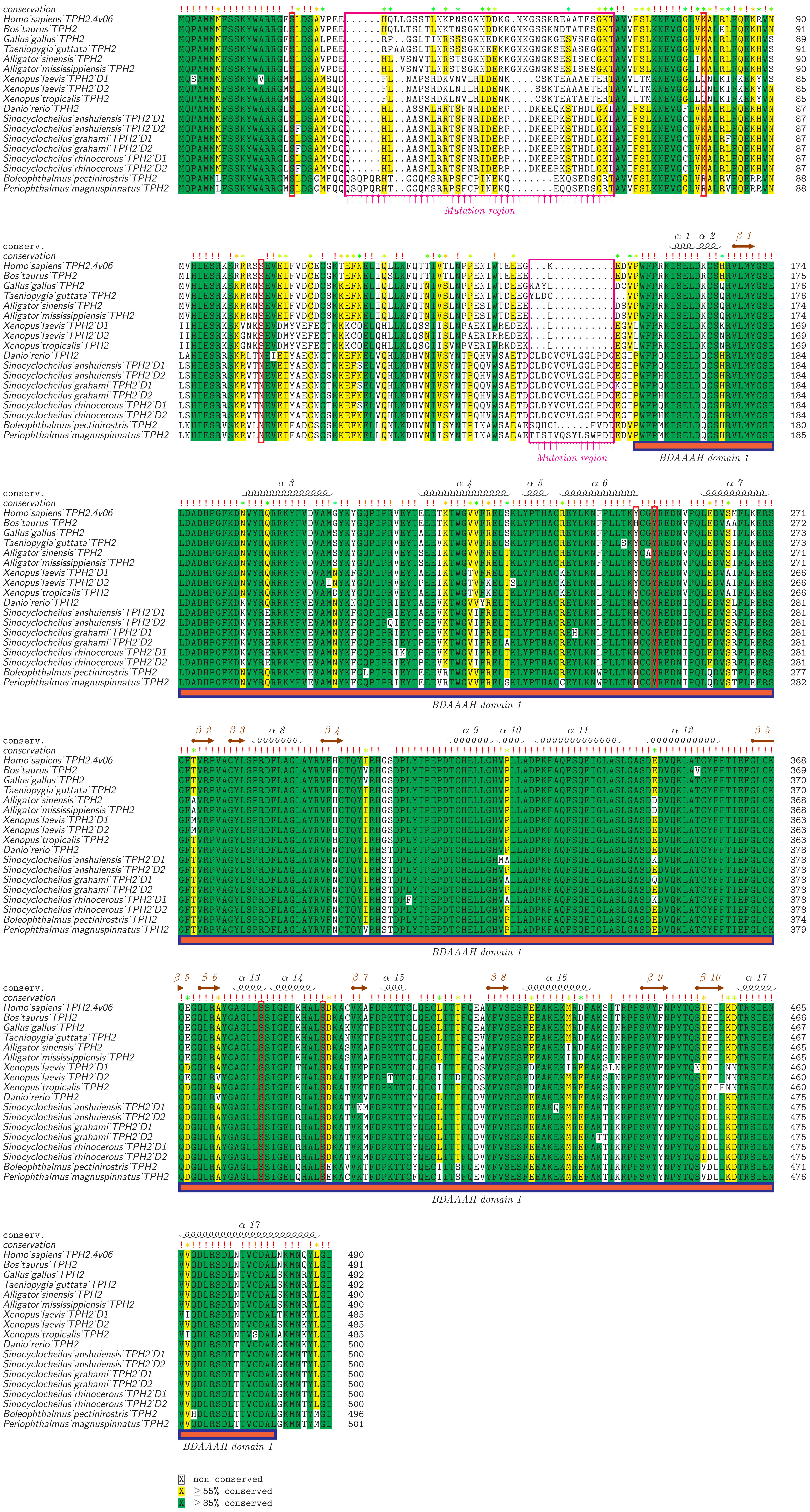
conservation
Homo sapiens TPH2.4v06
Bos taurus TPH2
Gallus gallus TPH2
Taeniopygia guttata TPH2
Alligator sinensis TPH2
Alligator mississippiensis TPH2
Xenopus laevis TPH2 D1
Xenopus laevis TPH2 D2
Xenopus tropicalis TPH2
Danio rerio TPH2
Sinocyclocheilus anshuiensis TPH2 D1
Sinocyclocheilus anshuiensis TPH2 D2
Sinocyclocheilus grahami TPH2 D1
Sinocyclocheilus grahami TPH2 D2
Sinocyclocheilus rhinocerous TPH2 D1
Sinocyclocheilus rhinocerous TPH2 D2
Boleophthalmus pectinirostris TPH2
Periophthalmus magnuspinnatus TPH2
Mutation region
conserv.
BDAAAH domain 1
X non conserved
X ≥55% conserved
X ≥85% conserved
