# Supplementary material for: Molecular Evolution of Tryptophan Hydroxylases in Vertebrates: A Comparative Genomic Survey
Source: Genes (Basel). 2019 Mar 8;10(3):203. doi: 10.3390/genes10030203 (PMC6470480; doi:10.3390/genes10030203)
Supplement: Supplementary file 1 [file genes-10-00203-s001.zip › Supplementary_Materials/Supplementary Materials.docx]

**Table S1.** GenBank ID of the selected 70 vertebrate genomes.

| **Class** | **Common Name** | **Species Name** | **GenBank ID** |
| --- | --- | --- | --- |
| **Mammals** | cattle | *Bos taurus* | GCF_000003055.6 |
|  | Chinese tree shrew | *Tupaia chinensis* | GCF_000334495.1 |
|  | crab eating macaque | *Macaca fascicularis* | GCF_000364345.1 |
|  | giant panda | *Ailuropoda melanoleuca* | GCF_000004335.2 |
|  | damara mole rat | *Fukomys damarensis* | GCF_000743615.1 |
|  | house mouse | *Mus musculus* | GCF_000001635.25 |
|  | human | *Homo sapiens* | GCF_000001405.37 |
|  | minke whale | *Balaenoptera acutorostrata* | GCF_000493695.1 |
|  | norway rat | *Rattus norvegicus* | GCF_000001895.5 |
|  | pygmy chimpanzee | *Pan paniscus* | GCF_000258655.2 |
|  | sperm whale | *Physeter catodon* | GCF_002837175.1 |
| **Birds** | adelie penguin | *Pygoscelis adeliae* | GCF_000699105.1 |
|  | African ostrich | *Struthio camelus* | GCF_000698965.1 |
|  | American crow | *Corvus brachyrhynchos* | GCF_000691975.1 |
|  | bald eagle | *Haliaeetus leucocephalus* | GCF_000737465.1 |
|  | budgerigar | *Melopsittacus undulatus* | GCF_000238935.1 |
|  | chicken | *Gallus gallus* | GCA_000002315.4 |
|  | common cuckoo | *Cuculus canorus* | GCF_000709325.1 |
|  | crested ibis | *Nipponia nippon* | GCF_000708225.1 |
|  | emperor penguin | *Aptenodytes forsteri* | GCF_000699145.1 |
|  | golden-collared manakin | *Manacus vitellinus* | GCF_001715985.1 |
|  | Tibetan ground-tit | *Pseudopodoces humilis* | GCF_000331425.1 |
|  | hoazin | *Opisthocomus hoazin* | GCA_000692075.1 |
|  | killdeer | *Charadrius vociferus* | GCF_000708025.1 |
|  | little egret | *Egretta garzetta* | GCF_000687185.1 |
|  | mallard | *Anas platyrhynchos* | GCF_000355885.1 |
|  | medium ground finch | *Geospiza fortis* | GCF_000277835.1 |
|  | peregrine falcon | *Falco peregrinus* | GCF_000337955.1 |
|  | zebra finch | *Taeniopygia guttata* | GCF_000151805.1 |
| **Reptiles** | American alligator | *Alligator mississippiensis* | GCF_000281125.3 |
|  | Australian crocodile | *Crocodylus porosus* | GCF_001723895.1 |
|  | Chinese alligator | *Alligator sinensis* | GCF_000455745.1 |
|  | gharial | *Gavialis gangeticus* | GCF_001723915.1 |
|  | gekko | *Gekko japanicus* | GCF_001447785.1 |
|  | green anole | *Anolis carolinensis* | GCF_000090745.1 |
|  | green sea turtle | *Chelonia mydas* | GCF_000344595.1 |
|  | king cobra | *Ophiophagus_hannah* | GCA_000516915.1 |
|  | painted turtle | *Chrysemys picta bellii* | GCF_000090745.1 |

**Table S1.** Continued.

| **Class** | **Common Name** | | **Species Name** | | **GenBank ID** | |
| --- | --- | --- | --- | --- | --- | --- |
| **Amphibians** | | African clawed frog | | *Xenopus laevis* | | GCF_001663975.1 |
|  | | tropical clawed frog | | *Xenopus tropicalis* | | GCF_000004195.3 |
|  | | Xizang plateau frog | | *Nanorana parkeri* | | GCF_000935625.1 |
| **Actinopterygii** | | American paddlefish | | *Polyodon spathula* | | Unpublished |
|  | | Asian arowana | | *Scleropages formosus* | | GCF_001624265.1 |
|  | | Atlantic salmon | | *Salmo salar* | | GCF_000233375.4 |
|  | | BP | | *Boleophthalmus pectinirostris* | | GCF_000788275.1 |
|  | | burton mouthbrooder | | *Astatotilapia burtoni* | | GCF_000239415.1 |
|  | | channel catfish | | *Ictalurus punctatus* | | [GCF_001660625.1](https://www.ncbi.nlm.nih.gov/assembly/763271) |
|  | | Chinese sturgeon | | *Acipenser sinensis* | | Unpublished |
|  | | fugu | | *Takifugu rubripes* | | GCF_000180615.1 |
|  | | PM | | *Periophthalmus magnuspinnatus* | | GCA_000787105.1 |
|  | | Sa | | *Sinocyclocheilus anshuiensis* | | GCF_001515605.1 |
|  | | grass carp | | *Ctenopharyngodon idellus* | | [PRJEB5920](http://www.ncbi.nlm.nih.gov/bioproject/?term=PRJEB5920) |
|  | | large yellow croaker | | *Larimichthys crocea* | | GCF_000972845.1 |
|  | | medaka | | *Oryzias latipes* | | GCF_000313675.1 |
|  | | mexican tetra | | *Astyanax mexicanus* | | GCF_000372685.1 |
|  | | Kanglang fish | | *Anabarilius grahami* | | GCA_003731715.1 |
|  | | northern pike | | *Esox lucius* | | GCA_000721915.3 |
|  | | nyerrrei cichlid | | *Pundamilia nyererei* | | GCF_000239375.1 |
|  | | platyfish | | *Xiphophorus maculatus* | | GCF_000241075.1 |
|  | | rainbow trout | | *Oncorhynchus mykiss* | | GCF_002163495.1 |
|  | | red-bellied piranha | | *Pygocentrus nattereri* | | GCF_001682695.1 |
|  | | seahorse | | *Hippocampus comes* | | GCF_001891065.1 |
|  | | Sg | | *Sinocyclocheilus grahami* | | GCF_001515645 |
|  | | spotter gar | | *Lepisosteus oculatus* | | GCF_000242695.1 |
|  | | Sr | | *Sinocyclocheilus rhinocerous* | | GCF_001515625 |
|  | | stickleback | | *Gasterosteus aculeatus* | | GCA_000180675.1 |
|  | | tilapia | | *Oreochromis niloticus* | | GCF_001858045.1 |
|  | | tongue sole | | *Cynoglossus semilaevis* | | GCF_000523025.1 |
|  | | zebrafish | | *Danio rerio* | | GCF_000002035.4 |
| Chondrichthyes | | elephant shark | | *Callorhynchus milii* | | GCF_000165045.1 |

**Table S2.** Queries used for extraction of *tph* genes from the examined 70 vertebrate species.

| **Gene** | **Class** | **Species for Queries** | **Queries Length** | **Protein Sequence** |
| --- | --- | --- | --- | --- |
| *tph1* | Mammals | *Homo sapiens* | 444 | NP_004170.1 |
| *tph1* | Birds | *Gallus gallus* | 445 | NP_990287.1 |
| *tph1* | Reptiles | *Chrysemys picta bellii* | 481 | XP_005308740.1 |
| *tph1* | Amphibians | *Xenopus laevis* | 481 | NP_001080923.1 |
| *tph1a* | Actinopterygii | *Danio rerio* | 483 | NP_840091.2 |
| *tph1b* | Actinopterygii | *Danio rerio* | 480 | AAI54121.1 |
| *tph2* | Mammals | *Homo sapiens* | 490 | NP_775489.2 |
| *tph2* | Birds | *Gallus gallus* | 489 | NP_001001301.1 |
| *tph2* | Reptiles | *Chrysemys picta bellii* | 491 | XP_005305157.3 |
| *tph2* | Amphibians | *Homo sapiens* | 490 | NP_775489.2 |
| *tph2* | Actinopterygii | *Danio rerio* | 500 | AAT38216 |

**Table S3.** Copy number variation in *tph* genes in the selected vertebrate genomes.

| **Class** | **Common Name** | **Species Name** | ***tph1* (*tph1a*)** | ***tph1b*** | ***tph2*** |
| --- | --- | --- | --- | --- | --- |
| **Mammals** | cattle | *Bos taurus* | 1 | - | 1 |
|  | chinese tree shrew | *Tupaia chinensis* | 1 | - | 1 |
|  | crab eating macaque | *Macaca fascicularis* | 1 | - | 1 |
|  | giant panda | *Ailuropoda melanoleuca* | 1 | - | 1 |
|  | damara mole rat | *Fukomys damarensis* | 1 | - | 1 |
|  | house mouse | *Mus musculus* | 1 | - | 1 |
|  | human | *Homo sapiens* | 1 | - | 1 |
|  | minke whale | *Balaenoptera acutorostrata* | 1 | - | 1 |
|  | norway rat | *Rattus norvegicus* | 1 | - | 1 |
|  | pygmy chimpanzee | *Pan paniscus* | 1 | - | 1 |
|  | sperm whale | *Physeter catodon* | 1 | - | 1 |
| **Birds** | adelie penguin | *Pygoscelis adeliae* | 1 | - | 1 |
|  | African ostrich | *Struthio camelus* | 1 | - | 1 |
|  | American crow | *Corvus brachyrhynchos* | 1 | - | 1 |
|  | bald eagle | *Haliaeetus leucocephalus* | 1 | - | 1 |
|  | budgerigar | *Melopsittacus undulatus* | 1 | - | 1 |
|  | chicken | *Gallus gallus* | 1 | - | 1 |
|  | common cuckoo | *Cuculus canorus* | 1 | - | 1 |
|  | crested ibis | *Nipponia nippon* | 1 | - | 1 |
|  | emperor penguin | *Aptenodytes forsteri* | 1 | - | 1 |
|  | golden-collared manakin | *Manacus vitellinus* | 1 | - | 1 |
|  | Tibetan ground-tit | *Pseudopodoces humilis* | 1 | - | 1 |
|  | hoazin | *Opisthocomus hoazin* | 1 | - | 1 |
|  | killdeer | *Charadrius vociferus* | 1 | - | 1 |
|  | little egret | *Egretta garzetta* | 1 | - | 1 |
|  | mallard | *Anas platyrhynchos* | 1 | - | 1 |
|  | medium ground finch | *Geospiza fortis* | 1 | - | 1 |
|  | peregrine falcon | *Falco peregrinus* | 1 | - | 1 |
|  | zebra finch | *Taeniopygia guttata* | 1 | - | 1 |
| **Reptiles** | American alligator | *Alligator mississippiensis* | 1 | - | 1 |
|  | Australian crocodile | *Crocodylus porosus* | 1 | - | 1 |
|  | Chinese alligator | *Alligator sinensis* | 1 | - | 1 |
|  | gharial | *Gavialis gangeticus* | 1 | - | 1 |
|  | gekko | *Gekko japanicus* | 1 | - | 1 |
|  | green anole | *Anolis carolinensis* | 1 | - | 1 |
|  | green sea turtle | *Chelonia mydas* | 1 | - | 1 |
|  | king cobra | *Ophiophagus_hannah* | 1 | - | 1 |
|  | painted turtle | *Chrysemys picta bellii* | 1 | - | 1 |

**Table S3.** Continued.

| **Class** | **Common Name** | **Species Name** | ***tph1***  **(*tph1a*)** | ***tph1b*** | ***tph2*** |
| --- | --- | --- | --- | --- | --- |
| **Amphibian** | African clawed frog | *Xenopus laevis* | 2 | - | 2 |
|  | tropical clawed frog | *Xenopus tropicalis* | 1 | - | 1 |
|  | Xizang plateau frog | *Nanorana parkeri* | 1 | - | 1 |
| **Actinopterygii** | American paddlefish | *Polyodon spathula* | 2 | - | 2 |
|  | Asian arowana | *Scleropages formosus* | 1 | - | 1 |
|  | Atlantic salmon | *Salmo salar* | 1 | 2 | 1 |
|  | BP | *Boleophthalmus pectinirostris* | 1 | 1 | 1 |
|  | burton mouthbrooder | *Astatotilapia burtoni* | 1 | 1 | 1 |
|  | Channel catfish | *Ictalurus punctatus* | 1 | 1 | 1 |
|  | Chinese sturgeon | *Acipenser sinensis* | 8 | - | 5 |
|  | fugu | *Takifugu rubripes* | 1 | - | 1 |
|  | PM | *Periophthalmus magnuspinnatus* | 1 | 1 | 1 |
|  | grass carp | *Ctenopharyngodon idellus* | 1 | 1 | 1 |
|  | large yellow croaker | *Larimichthys crocea* | 1 | 1 | 1 |
|  | medaka | *Oryzias latipes* | 1 | 1 | 1 |
|  | mexican tetra | *Astyanax mexicanus* | 1 | 1 | 1 |
|  | Kanglang fish | *Anabarilius grahami* | 1 | 1 | 1 |
|  | northern pike | *Esox lucius* | 1 | - | 1 |
|  | nyerrrei cichlid | *Pundamilia nyererei* | 1 | 1 | 1 |
|  | platyfish | *Xiphophorus maculatus* | 1 | 1 | 1 |
|  | rainbow trout | *Oncorhynchus mykiss* | 1 | 2 | 1 |
|  | red-bellied piranha | *Pygocentrus Nattereri* | 1 | 1 | 1 |
|  | Sa | *Sinocyclocheilus anshuiensis* | 1 | 2 | 2 |
|  | seahorse | *Hippocampus comes* | 1 | 1 | 1 |
|  | Sg | *Sinocyclocheilus grahami* | 1 | 2 | 2 |
|  | spotter gar | *Lepisosteus oculatus* | 1 | - | 1 |
|  | Sr | *Sinocyclocheilus rhinocerous* | 1 | 2 | 2 |
|  | stickleback | *Gasterosteus aculeatus* | 1 | 1 | 1 |
|  | tilapia | *Oreochromis niloticus* | 1 | 1 | 1 |
|  | tongue sole | *Cynoglossus semilaevis* | 1 | - | 1 |
|  | zebrafish | *Danio rerio* | 1 | 1 | 1 |
| Chondrichthyes | elephant shark | *Callorhynchus milii* | 1 | - | 1 |

-: unidentified; ^1^ Tetraploid fishes; ^2^ Amphibious fishes.

**
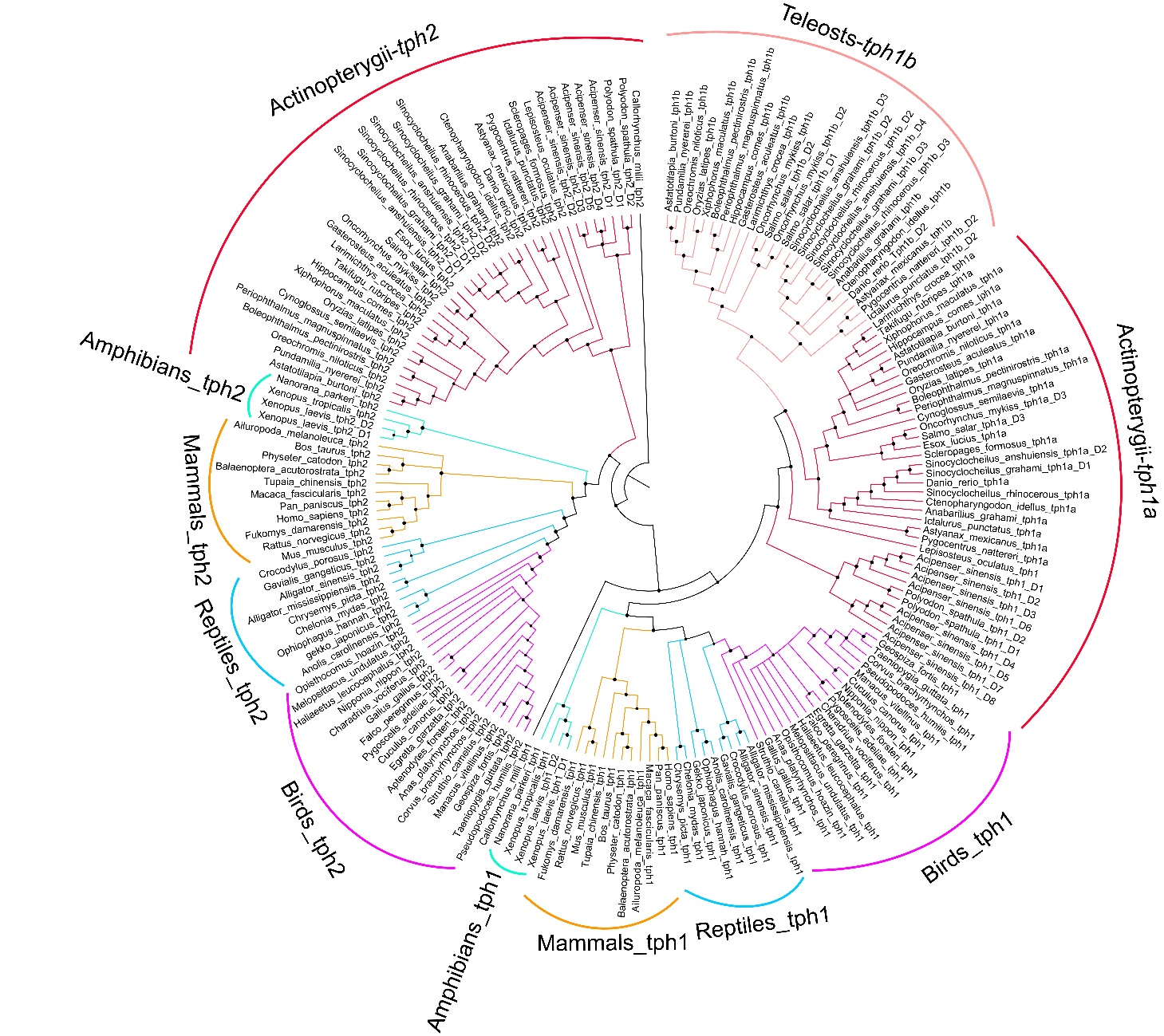
**

**Figure S1.** BI tree deduced from the 184 vertebrate TPH protein sequences.


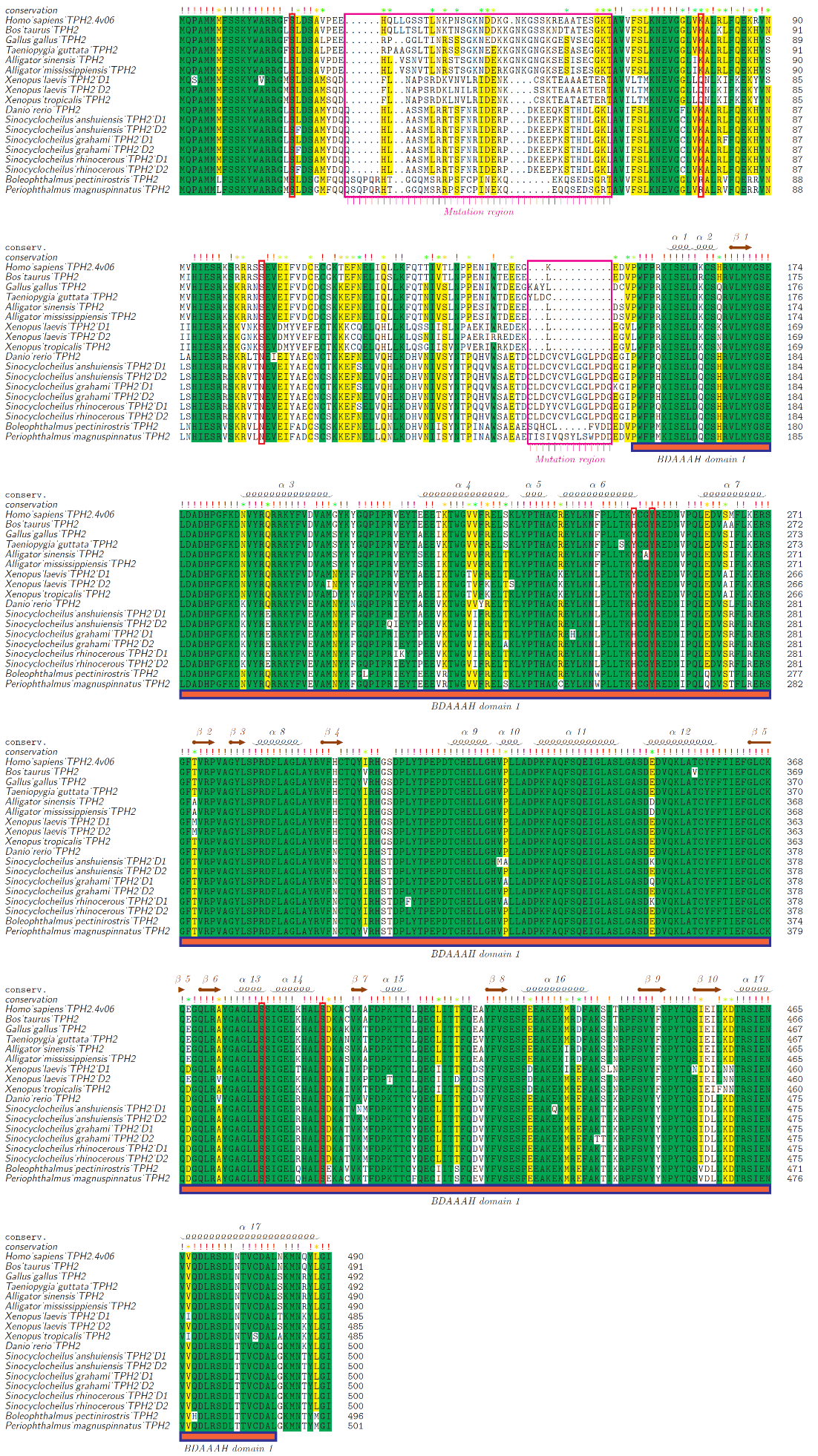


(a)


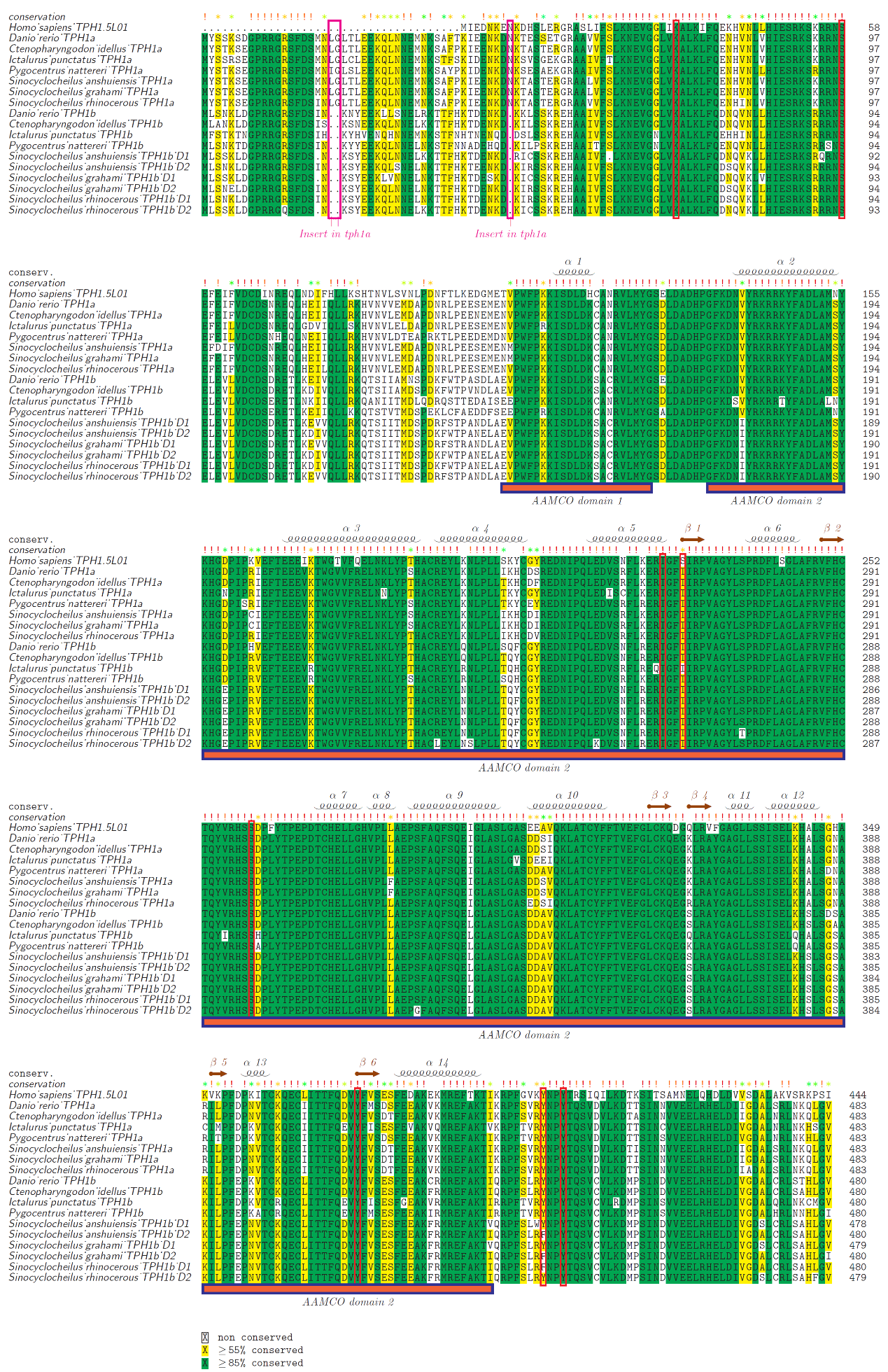


(b)


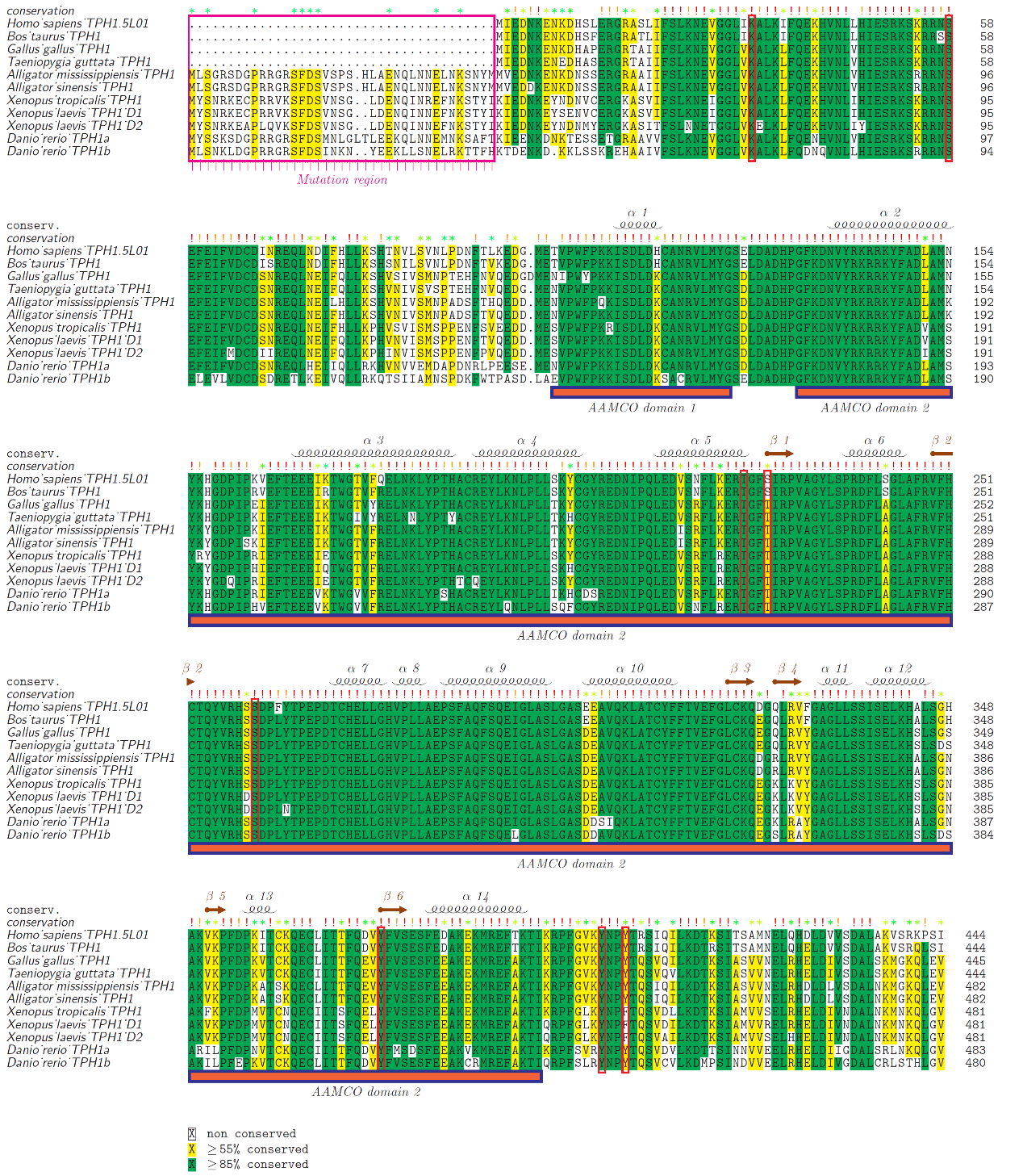
(c)

**F****igure S2.** Alignment and secondary structures of TPH protein sequences in additional vertebrate species. (**a**) TPH2 protein sequences from representative vertebrate species were aligned with the human TPH2 and its secondary structure template 4V06. (**b**) Sequence alignment between TPH1a and TPH1b in some representative teleost was provided for comparison. (**c**) TPH1 protein sequences from representative vertebrates were aligned with the human TPH1 and its secondary structure template 5L01. The mutation regions were marked with rose boxes and upper arrow in each figure. The red boxes denote the phosphorylation sites on the basis of the human templates.


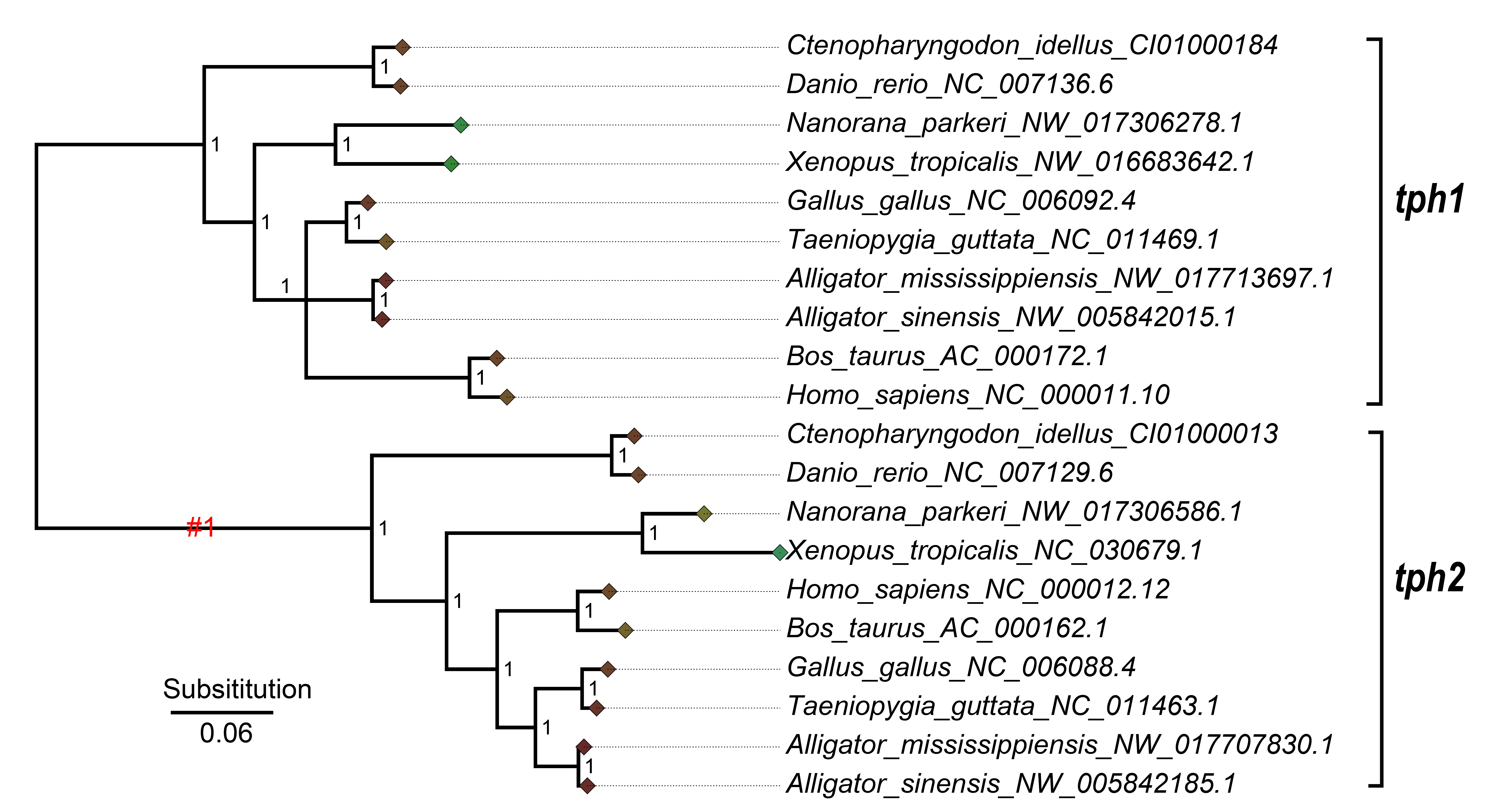


**Figure S3.** The BI tree of TPH1 and TPH2 proteins in additional vertebrate species for selection pressure analyses. #1 denotes the foreground of TPH2 clade. Numbers in the topology indicate the Bayesian posterior probabilities. Each diamond at the tip of every branch was colored based on its branch length, with a darker color to represent a shorter evolutionary branch.
