# Supplementary figures and images for: Molecular Evolution of Tryptophan Hydroxylases in Vertebrates: A Comparative Genomic Survey
Source: Genes (Basel). 2019 Mar 8;10(3):203. doi: 10.3390/genes10030203 (PMC6470480; doi:10.3390/genes10030203)

[illegible][illegible]

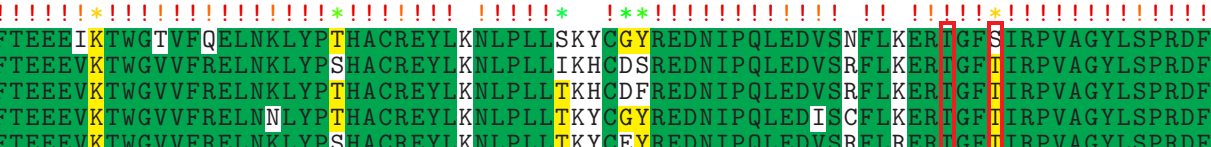

|   |                 |
|---|-----------------|
| X | non conserved   |
| X | ≥ 55% conserved |
| X | ≥ 85% conserved |

Supplement: Supplementary file 1 [file genes-10-00203-s001.zip › Supplementary_Materials/Figure S2-b.pdf]

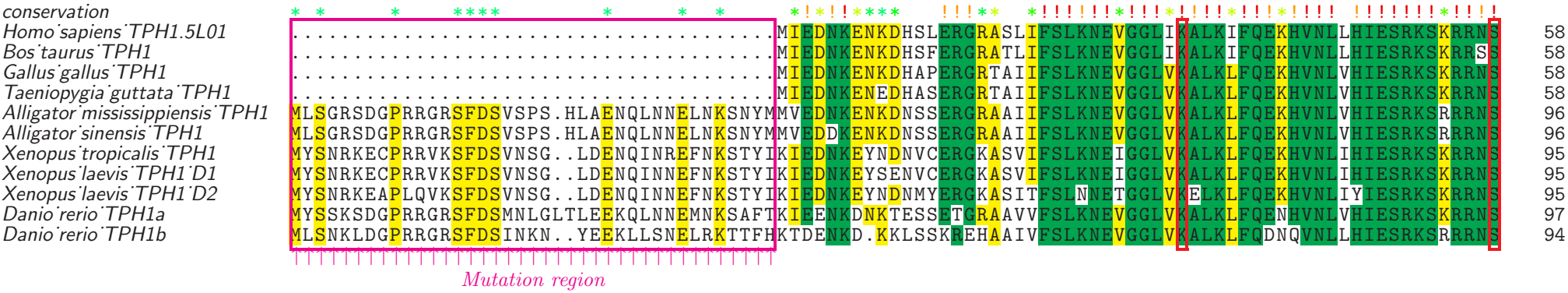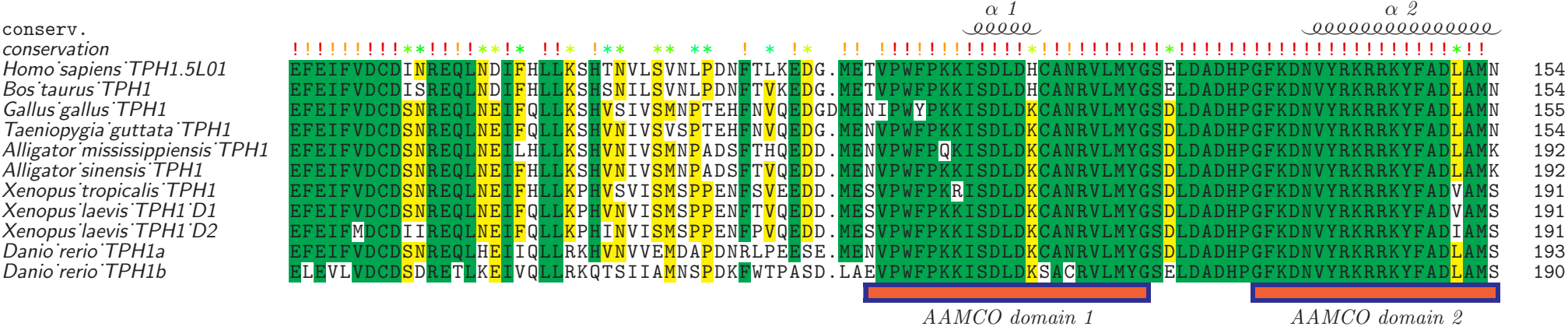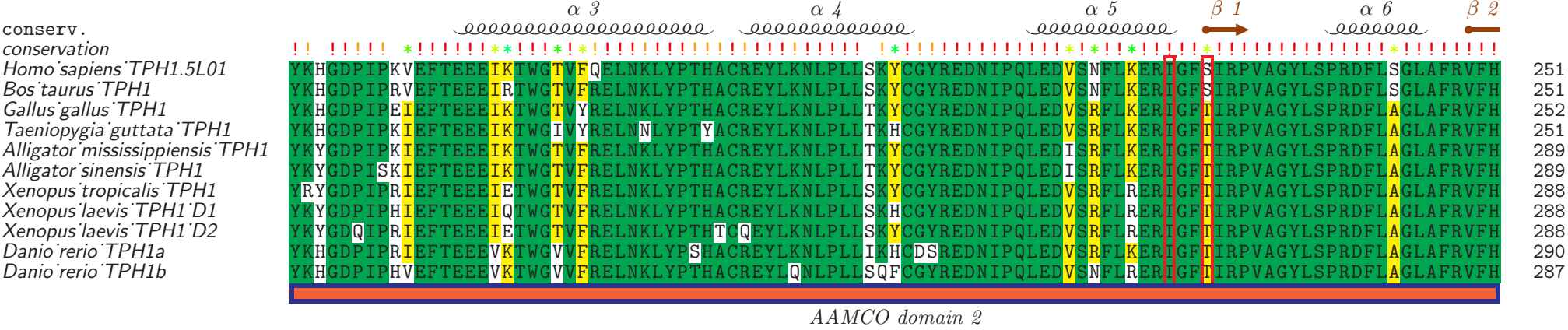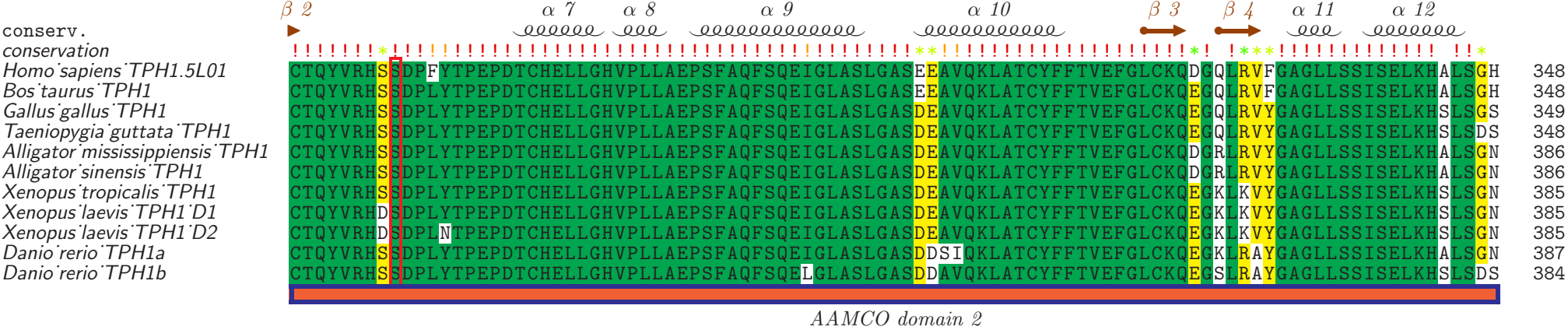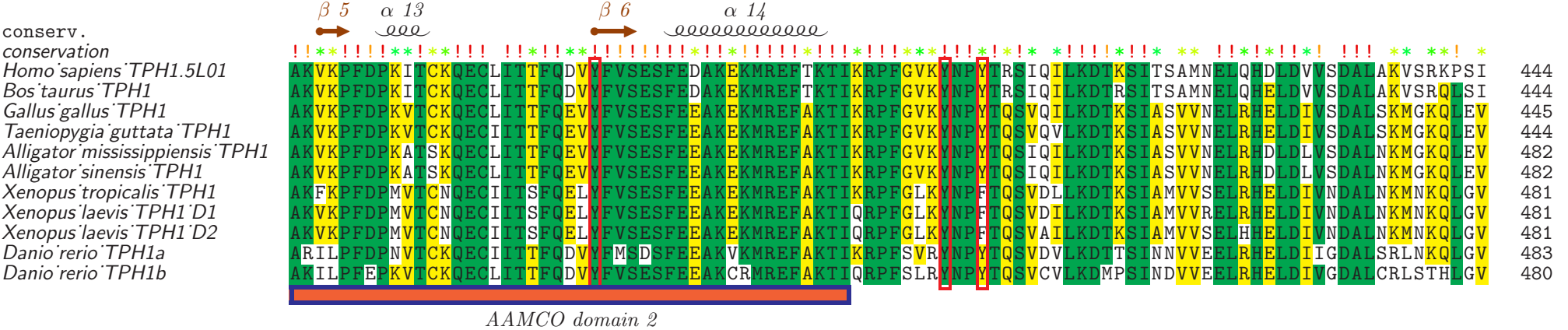

☐ non conserved

☒ ≥ 55% conserved

☑ ≥ 85% conserved

Supplement: Supplementary file 1 [file genes-10-00203-s001.zip › Supplementary_Materials/Figure S2-c.pdf]

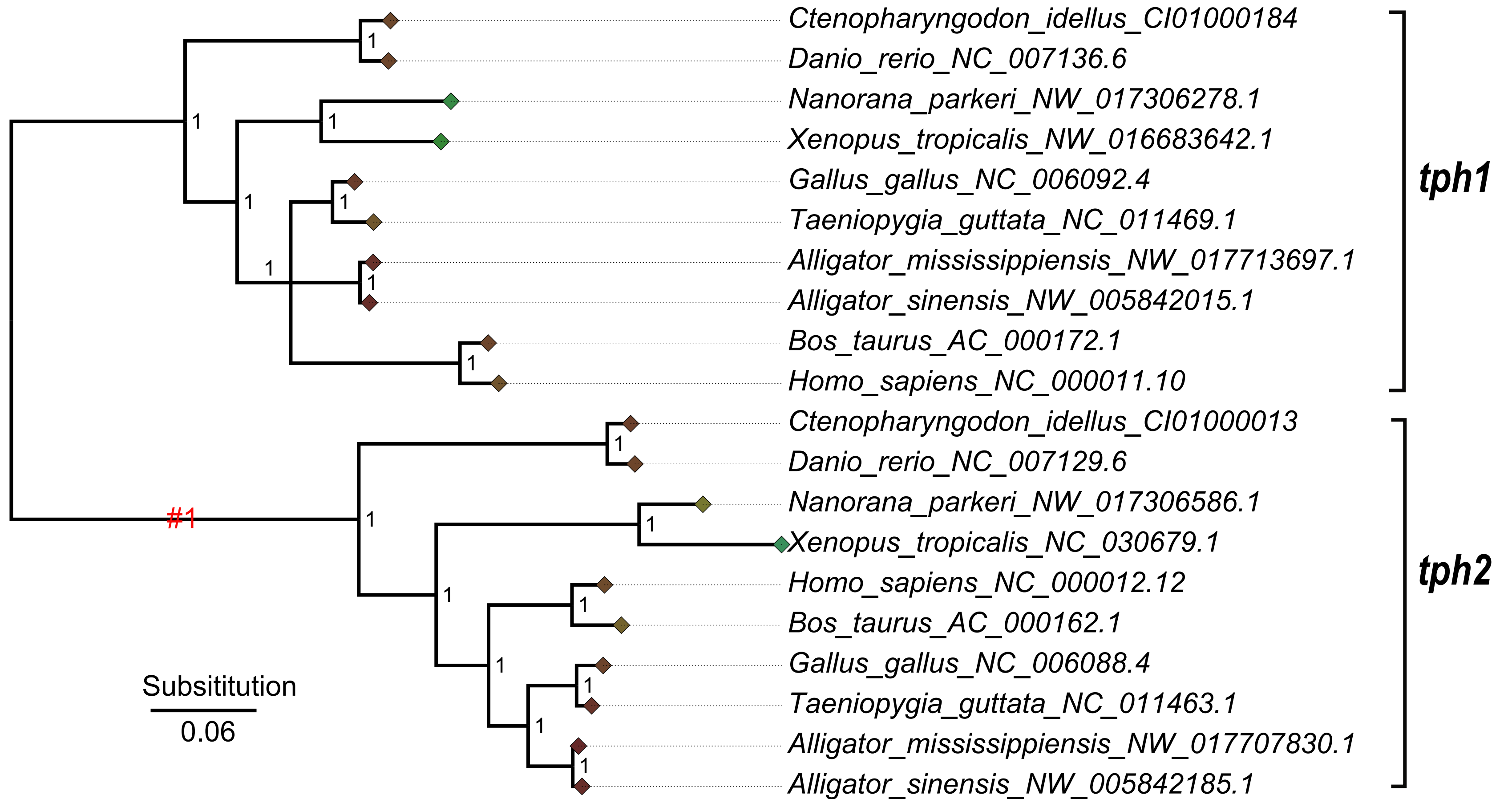

Supplement: Supplementary file 1 [file genes-10-00203-s001.zip › Supplementary_Materials/Figure S3.pdf]
